# Virulence Adaptation by Rice Planthoppers and Leafhoppers to Resistance Genes and Loci: A Review

**DOI:** 10.3390/insects15090652

**Published:** 2024-08-29

**Authors:** Finbarr G. Horgan

**Affiliations:** 1 EcoLaVerna Integral Restoration Ecology, Bridestown, Kildinan, T56 P499 County Cork, Ireland; f.horgan@ecolaverna.org; 2 Faculty of Agrarian and Forest Sciences, School of Agronomy, Catholic University of Maule, Casilla 7-D, Curicó 3349001, Chile; 3 Centre for Pesticide Suicide Prevention, University/BHF Centre for Cardiovascular Science, University of Edinburgh, Edinburgh EH16 4TJ, UK

**Keywords:** brown planthopper, endosymbionts, green leafhopper, host plant resistance, microbiome, yeast-like symbionts

## Abstract

**Simple Summary:**

Rice breeding programs have included traditional varieties and wild rice species as sources of resistance to planthoppers and leafhoppers, which are major insect pests of rice in Asia. However, herbivores can adapt to resistance (known as virulence adaptation), sometimes in as little as 2 years. Virulence adaptation is a major obstacle for deploying resistant varieties. This review assesses knowledge of the rates, consequences, and mechanisms of virulence adaptation and on possible strategies to delay adaptation in rice landscapes. The review indicates that virulence is a complex of responses to minor and major resistance factors. Of particular interest are adaptations to major rice resistance genes that are likely governed by corresponding herbivore virulence genes. Evidence suggests that pre-adapted individuals (called forerunners) with these virulence genes occur in planthopper populations at relatively low frequencies but can rapidly build up numbers in response to planted resistant rice. However, gradual shifts in virulence may also occur due to intra- and intergenerational improvements in the herbivores’ abilities to detoxify plant defense toxins, or to confound defense response networks. These may involve gradual changes in the expression of genes involved in successful herbivore attacks and may be associated with the herbivores or their endosymbionts. Several research gaps remain. In particular, there is a need for increased attention to practical, ecologically based deployment strategies that delay adaptation.

**Abstract:**

In recent decades, research on developing and deploying resistant rice has accelerated due to the availability of modern molecular tools and, in particular, advances in marker-assisted selection. However, progress in understanding virulence adaptation has been relatively slow. This review tracks patterns in virulence adaptation to resistance genes (particularly *Bph1*, *bph2*, *Bph3*, and *bph4*) and examines the nature of virulence based on selection experiments, responses by virulent populations to differential rice varieties (i.e., varieties with different resistance genes), and breeding experiments that interpret the genetic mechanisms underlying adaptation. The review proposes that varietal resistance is best regarded as a combination of minor and major resistance traits against which planthoppers develop partial or complete virulence through heritable improvements that are reversable or through evolutionary adaptation, respectively. Agronomic practices, deployment patterns, and herbivore population pressures determine the rates of adaptation, and there is growing evidence that pesticide detoxification mechanisms can accelerate virulence adaptation. Research to delay adaptation has mainly focused on gene pyramiding (i.e., including ≥ two major genes in a variety) and multilines (i.e., including ≥ two resistant varieties in a field or landscape); however, these strategies have not been adequately tested and, if not managed properly, could inadvertently accelerate adaptation compared to sequential deployment. Several research gaps remain and considerable improvements in research methods are required to better understand and manage virulence adaptation.

## 1. Introduction

Planthoppers and leafhoppers are regarded among the most damaging insect herbivores of rice in Asia [1,2,3,4,5,6,7]. Records of damage to rice in Korea and Japan by migratory planthoppers date back thousands of years [8]; however, it was only since the beginning of the Green Revolution in the 1960s, that largescale, widespread, and persistent outbreaks of the brown planthopper (*Nilaparvata lugens* [BPH]) and whitebacked planthopper (*Sogatella furcifera* [WBPH]) became a serious constraint to rice production: between the late 1960s and mid-1970s, largescale outbreaks of these two species were documented for the first time in Indonesia, the Philippines, and several states (e.g., Kerala, Tamil Nadu, Coimbatore) in India [8]. In other regions, outbreaks occurred at a greater scale than had previously been recorded (e.g., Taiwan [9], Sri Lanka [10], Thailand [11]). Researchers associated these outbreaks with the introduction and widespread adoption by farmers of modern high-yielding, semi-dwarf rice varieties (HYV) and heavy applications of fertilizers [8,12,13,14]; however, it is now widely accepted that excessive pesticide use ultimately induced many of these planthopper outbreaks [15,16,17,18]. New viral diseases were also associated with the early (1960s–1970s) outbreaks: the rice ragged stunt virus (RSV), transmitted by BPH, first infested rice over large areas during the mid-late 1970s [19,20,21]. At about the same time, leafhoppers (*Nephotettix* spp.) became problematic in HYVs as vectors of tungro viruses [22,23,24].

In response to outbreaks, traditional rice varieties and landraces were screened by research institutes across Asia for their resistance to planthoppers and leafhoppers [25,26,27]. The most resistant varieties (largely based on screening with planthopper populations from the Philippines) were mainly from India and Sri Lanka. These were integrated into early resistance breeding programs, particularly at the International Rice Research Institute (IRRI), and became the focal varieties for resistance research and a basis for the development of resistant rice varieties for several decades, and indeed until the present [28,29,30,31]. More recently, resistant rice lines with genes introgressed from wild rice species have played an increasingly greater role in the development of commercial, planthopper-resistant rice varieties, particularly for hybrid rice in China [28,29,32].

Soon after the first resistant rice varieties were released in Asia in the 1970s and 1980s, it became apparent that rice planthoppers and leafhoppers had the potential to quickly adapt to and overcome (known as ‘virulence adaptation’) deployed resistance [33,34,35,36]. A rapid adaptation by BPH to rice lines with resistance from wild rice species has also been documented [37]. As planthoppers and leafhoppers adapt to resistant rice varieties, the related resistance genes may lose their utility for future breeding programs. For example, planthoppers have already adapted to widely deployed resistance loci, including *Bph1*, *bph2*, *Wbph1*, and *Wbph2*, across South and Southeast Asia [38,39,40], thereby largely excluding these resistance sources from future breeding programs. This is exacerbated where migratory planthoppers and leafhoppers carry virulence to regions where the corresponding resistance genes may not yet have been deployed [41,42,43].

Planthopper outbreaks subsided throughout the 1990s, perhaps due to a reduction in pesticide use encouraged by the Food and Agriculture Organization (FAO) of the United Nations and other international programs [44,45]; as a consequence, research attention to planthopper and leafhopper resistance in rice waned [46]. However, since about 2005, major outbreaks of BPH and WBPH again occurred in China, Vietnam, Indonesia, Thailand, and India, reaching their maximum extent and severity between 2009 and 2013 [2,6,7]. These recent outbreaks, together with modern advances in molecular biology and plant breeding, encouraged renewed research attention into anti-herbivore resistance in rice with a greater emphasis on using wild rice species as sources of resistance and on pyramiding two or more genes to increase the strength of resistance. Currently, over 80 gene loci and several quantitative trait loci (QTLs) have been associated with rice resistance to planthoppers and leafhoppers [28,29,30,31]. A number of modern varieties with recognized resistance have also been released through national breeding programs, albeit at a relatively slow pace (i.e., compared to research on gene discovery) [47].

Whereas recent research has led to a proliferation of studies related to gene discovery, molecular breeding techniques, gene regulation, and proteomics, and the molecular mechanisms underlying resistance [28,29,30,31,32,48], considerably less attention has been given to issues of herbivore adaptation and gene deployment strategies that might prolong varietal resistance [49]. The sequence of events since the beginning of the millennium, including a phenomenal increase in pesticide trade and use since 2000 [50], largescale planthopper outbreaks across Asia [7], an overwhelming emphasis on host plant resistance as the main research response to these outbreaks [46,50], and the widespread and efficient sharing of resistance genes/sources internationally [47,51], suggest that history might be repeated and that rare and useful resistance genes, which should be considered a public good, will be lost for posterity.

This review draws together historical and recent information on virulence adaptation by planthoppers and leafhoppers to resistance genes and loci. The review first examines the frequency of resistance to planthopper and leafhopper populations and proposes that resistance genes should be regarded as a rare genetic resource that must be responsibly managed. It then compiles observations from field reports of virulence adaptation and damage after the widescale deployment of resistant varieties, and from laboratory studies of planthopper and leafhopper selection, to address adaptation rates and the nature of adaptation. Furthermore, it reviews studies of relative herbivore responses to resistant rice that indicate possible factors that facilitate adaptation and likely trade-offs associated with adaptation. Finally, the review examines the potential mechanisms underlying adaptation and possible methods to slow adaptation and, thereby, to conserve resistance sources. It is hoped that the review will form a basis for future research into virulence adaptation in rice and, in particular, will encourage the development of further ecologically based deployment strategies to prolong the utility of resistance genes.

## 2. Literature Review

A literature search was conducted using Web of Science (1970–July 2024) and Google Scholar, applying the terms ‘*Nilaparvata*’, ‘*Sogatella*’, *Laodelphax*’, ‘*Nephotettix*’, ‘*Recilia*’, or ‘*Tagosodes*’, and ‘virulence’, ‘adaptation’, ‘biotype’, ‘pyramid*’, or ‘durab*’. The initial search retrieved 286 documents, of which 64 were omitted because they made only cursory reference to virulence adaptation, were not accessible, or contained duplicated information (=222); a second search was made by reviewing the indexes of all volumes of International Rice Research Notes (IRRN) for relevant articles and all retrieved documents were used to expand the search and accumulate other relevant documents; this retrieved a further 65 documents (=287). To assess the prominence of rice resistance genes, a further search was made in Google Scholar using the insect genus names listed above with the terms ‘resistance’ and ‘screening’; the retrieved documents were assessed and only those describing large screening programs were considered (=43); recent screening studies that possibly duplicated rice genotypes were omitted (total = 330 documents).

## 3. Results and Discussion

### 3.1. Resistance Genes—A Rare Genetic Resource

A number of recent reviews present detailed lists of potential QTLs, gene loci, and genes associated with rice resistance to planthoppers and leafhoppers. Dozens of gene loci have now been identified; however, only recently have some of these loci been investigated to reveal one or more associated genes [28,29,30,31]. Many of the currently recognized resistance loci are the result of research that first began in the 1960s with the largescale screening of 100s or 1000s of rice varieties at research centers throughout Asia (noted exceptions are genes associated with the ovicidal response [52,53] that occurs in late-stage rice and is not possible to detect in standard bulk screening tests [54,55]) (Table 1). These centers generally applied a simple preference test known as the standard seedling seedbox test (SSST) to identify resistant materials, but sometimes also included more detailed bioassays (i.e., survival bioassays, honeydew tests, field screening, etc.) as susceptible rice lines were eliminated from screening programs and the numbers of potentially useful lines were reduced (see Figures S1 and S2 for further details of screening tests) [46,54,55].

Table 1 is a summary of the results from some published, mainly largescale screening studies. Based on the combined results of several studies, <10% of cultivated rice varieties demonstrate at least moderate resistance to BPH (Table 1). Most of these screened varieties have been traditional varieties or landraces available through national and international germplasm banks. Some rice types, for example *japonica* varieties, Tong-Il varieties, and South Asian scented varieties, have little or no seedling resistance to BPH [56,62,88]. Less than 5% of screened germplasm (including wild rice accessions) displayed strong resistance to BPH (i.e., standard evaluation system (SES) ≤ 3).

A greater proportion of screened materials (25–38%) were moderately resistant to WBPH, the small brown planthopper (SBPH), or leafhoppers (Table 1). In the case of WBPH and SBPH, about 10% of screened varieties had high levels of resistance. Rice resistance against the South American delphacid planthopper (*Tagosodes orizicola*) appears to be relatively common (ca 60% of varieties were at least moderately resistant) with ca 20% of accessions having high levels of resistance. Rice resistance to the zig-zag leafhopper (ZLH) is uncommon (<3%). In contrast to cultivated rice, a number of studies that screened wild rice species for resistance against Asian planthoppers and leafhoppers indicate a generally high incidence (38–95%) of often strong resistance across accessions (Table 1). High resistance to WBPH among wild rice accessions occurs among ca 10% of screened materials [71]. Although Table 1 might suggest that a large number of resistance genes have yet to be discovered that can be added to the ca 80 gene loci already identified [28,29,31], several points, as raised in the following paragraphs, should be considered.

Planthoppers and leafhoppers, in particular monophagous and oligophagous species, demonstrate regional preferences for locally prevalent varieties [42,89,90]. Such ‘preferences’ can be associated with a lower fitness on novel varieties to which the planthoppers or leafhoppers have never been exposed, even where the variety possesses no major resistance genes. Adaptation to novel host varieties can take several generations [37,89,91,92,93]. Such ‘apparent resistance’ is often indistinguishable from moderately resistant varieties linked to major genes in screening tests, and may include what was formerly regarded as weak ‘horizontal resistance’ governed by polygenic mechanisms (i.e., influenced by two or more genes and displaying often high environmental plasticity), which are unsuitable for resistance breeding programs [59]. Distinguishing between the ‘feeding inexperience’ or ‘naivety’ of the herbivore population and ‘true resistance’ that is associated with major resistance genes (i.e., ‘avirulence’ in the herbivore population) can be difficult without multi-generational screening (see below). For example, Rezaul Karim and Pathak (1979) [94] reported that of the 473 rice varieties with resistance to the green leafhopper (GLH) at IRRI and largely derived from South Asian sources, only 10 were resistant to GLH at the Bangladesh Rice Research Institute; furthermore, the variety IR24, for which no resistance genes/loci have been identified [95], often appears resistant to BPH in SSSTs from South Asia; and TN1, which is a popular susceptible check used during screening in Asia, appears resistant to Australian populations of BPH [38,39,91,96].

Given that varieties from the same regions are likely to have similar pedigrees, the actual number of resistance genes underlying observed resistance will be much smaller than the number of identified resistance sources. Furthermore, because of the dominance of the SSST during phenotyping and gene discovery, most planthopper resistance genes will represent the same or similar resistance mechanisms (usually expressed in seedlings and directed against feeding nymphs) [46,54]. Furthermore, many resistance loci, including some identified from wild rice species, are clustered around specific regions of the rice genome (i.e., cluster A on chromosome 12, clusters B and D on chromosome 4, and cluster C on chromosome 6) [28]. This suggests that different loci may occasionally represent the same resistance genes (with the same or different alleles) but from different rice lines (e.g., see Zhao et al., 2016 [97]). There are already several cases of redundant naming for loci (that contain the same genes) identified at different research centers, using different source materials, or using different test herbivores [28,32,98]. For these reasons, the adaptation by planthoppers and leafhoppers to one identified gene locus can often lead to the loss of a second locus derived from a different source [99,100,101,102,103].

Finally, many largescale screening studies were conducted during the 1970s–1990s at a time when planthopper and leafhopper populations are considered to have been less virulent than today [101,104,105,106,107]. Indeed, some gene discovery programs have used relict laboratory colonies (those collected and maintained during several years), or colonies initiated with a small number of wild-caught founders (usually <500), thereby identifying varieties or genes against which modern ‘wild’ or ‘field’ planthopper populations have already adapted [101,104,108]. For example, largescale, regional evaluations of over 30 donor varieties, representing 29 resistance gene loci, indicated that very few of the donors have maintained their seedling resistance against modern ‘field’ populations of planthoppers from South and Southeast Asia: Only ca 20% of the donor materials showed some resistance against >50% of BPH populations [38,39]. Based on these observations, it is likely that only ca 10–15 genes are currently useful for breeding programs against BPH, with similar numbers of identified loci currently useful against other planthoppers and leafhoppers [28,32].

### 3.2. Resistance Genes—A Non-Renewable Resource

If, as suggested above, major resistance genes are rare and likely to be overcome by herbivores, an important question is whether they can ever be renewed (i.e., that planthopper populations lose their virulence adaptations). Evidence indicates that the adaptation by BPH to regionally deployed genes, such as *Bph1* and *bph2*, has now remained fixed in BPH populations for several decades [101,104,105]. This suggests that adaptation to these genes has not incurred fitness penalties. However, it should be noted that BPH populations in many regions are also likely to have remained exposed to varieties with *Bph1* and *bph2*, particularly since the donor varieties were prominent in breeding programs throughout Asia in the 1980s and 1990s [109,110].

Varieties with resistance loci for *Bph3* (possibly containing the *Bph32* gene, see below) or *bph4* have also been widely deployed [89,99,111], although not to the same extent as varieties with *Bph1* and/or *bph2*; however, it is difficult to determine whether there has ever been sufficient selection pressure for field BPH populations to overcome *Bph3* and *bph4* at wide scales, and for virulence against the genes to ever become fixed [99]. Nevertheless, in the Solomon Islands, 60% of Bg379-5 (*Bph3*) had hopperburn (i.e., conspicuous patches of dead rice due to planthopper feeding) only 2 years after the variety was introduced [111], and there are records of planthoppers infesting IR62 (*Bph3*) in Mindanao (Philippines) 4–6 years after the variety was released [112]. Furthermore, a number of studies have indicated notably high levels of virulence among BPH populations from the Mekong Delta, Vietnam, against varieties with the *Bph3* and *bph4* gene loci [43,113,114,115,116] and, since the 1990s, an estimated 5–10% of BPH migrating into Northeast Asia have been virulent against *Bph3* and *bph4* [101,105]. Few other resistance genes have been deliberately deployed in any region or, in cases where there has been relatively widescale deployment (e.g., *Bph14* and *Bph15* in China), there are still no field records of planthopper responses [29,117].

Despite long-term virulence, studies suggest that planthoppers and leafhoppers selected on resistant rice lines continue to feed inefficiently for several generations even after planthopper survival, growth, development, and oviposition on the resistant lines approach levels attained on susceptible varieties [37,118]; indeed, the widespread virulence of BPH to varieties with *Bph1* and *bph2* resistance, has also been associated with apparently inefficient feeding behaviors [119]. Even after planthoppers and leafhoppers have adapted to resistance, they apparently continue to prefer highly susceptible varieties in choice feeding and oviposition bioassays, but have a generally lower fecundity compared to avirulent populations, even on susceptible varieties [103,109,120,121,122]—although this may be due to inbreeding in caged populations [123]. Furthermore, compared to overwintering populations, the relatively delayed dominance among migrating planthoppers of individuals with virulence against *Bph1* and *bph2* suggests that there may be fitness costs in terms of migration potential associated with virulence [41]. Despite these observations, laboratory studies that have relaxed selection pressures by returning BPH to susceptible varieties for several generations indicate that virulence is not reversed [91,119,124]. This is also supported by evidence from relict BPH populations that remain virulent against resistance genes despite several generations in contained laboratory colonies on susceptible rice varieties [101,104,119,125,126].

In contrast to planthoppers, populations of leafhoppers appear to quickly lose virulence when returned to susceptible varieties (e.g., two [127] to seven [106] generations; but see Rapusas and Heinrichs (1982) and Horgan et al. (2018) [128,129]). Although aspects of leafhopper virulence against resistance genes have received relatively little research attention, there is evidence to suggest that leafhopper virulence is less stable than that of planthoppers, particularly where leafhoppers adapt to feed, but continue to lay few eggs on resistant varieties [93,130]. Overall, considering both planthoppers and leafhoppers, the sparce information that is available suggests that virulence can develop in localized pockets, and, although the trait may be retained over generations (particularly in planthoppers), widescale virulence will only develop in proportion to the area of gene deployment and will remain stable in the case of the continued deployment of the resistance genes, or other fitness advantages arising from the adaptation. Further experimental evidence is needed to help predict the likely outcomes from widescale resistance deployment for other and multiple genes.

### 3.3. The Nature of Virulence Adaptation

Knowledge accumulated from reports of adaptation by planthoppers and leafhoppers to resistant rice varieties and genes has come from a range of field observations and laboratory experiments. The information can be considered under four main categories: (1) historical evidence (1960s to early 2000s) derived from accumulated reports from agricultural extension officers and rice scientists throughout Asia; (2) the concurrent screening of relict planthopper populations using collections of rice varieties or near-isogenic lines (NILs) with known resistance genes; (3) a series of laboratory selection studies conducted using a range of planthopper and leafhopper species reared on different resistant varieties; and (4) the testing of resistant varieties for reactions to laboratory-selected, virulent populations. These will be discussed in the following subsections.

#### 3.3.1. Historical Evidence

**Figure 1:**
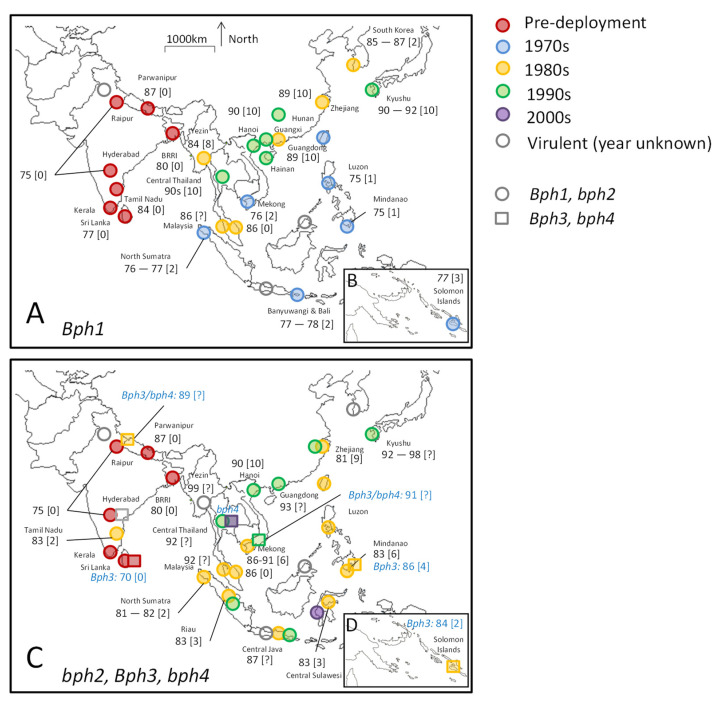
Adaptation by brown planthopper, *Nilaparvata lugens*, populations to resistant rice varieties with the *Bph1* (**A**,**B**), *bph2*, *Bph3*, and *bph4* (**C**,**D**) loci/genes. Numbers are the approximate years (i.e., 75 = 1975) when virulence was first noted against each indicated locus/gene in South and East Asia (**A**,**C**) and on the Solomon Islands (**B**,**D**). Note that dates associated with pre-deployment virulence in south Asia indicate the year that modern varieties with known resistance were first deployed. The numbers in brackets are the approximate years since largescale deployment of varieties with the indicated loci/genes and until widescale virulence was reported. Colored symbols indicate planthopper populations with apparent virulence before the deployment of modern HYVs (red), populations adapted to resistance genes in the 1970s (blue), 1980s (yellow), 1990s (green), and 2000s (purple), or with currently recognized virulence for which the approximate years of development are unknown (symbols with no color). Square symbols in (**C**,**D**) relate to the *Bph3* or *bph4* loci/genes as indicated. Further details are available in Table S1 including data sources [10,38,40,42,43,60,65,111,112,113,114,115,116,122,126,134,136,137,138,139,140,141,142,143,144,145,146,147,148,149,150,151,152,153,154,155,156,157,158,159,160,161,162,163,164,165,166,167,168,169,170,171,172,173,174,175,176,177,178,179,180,181,182,183,184,185,186,187].

Adaptation by BPH to the *Bph1* and *bph2* resistance genes is one of the best documented examples of crop–herbivore coevolution. Figure 1 maps the earliest reports of virulence adaptation by BPH to these genes at sites across South and Southeast Asia. The figure also indicates records of adaptation to *Bph3* and *bph4* by the planthopper. Three features of rice entomology and resistance research during the 1960s to early 2000s have made the information so extensive. These features are as follows: (1) the resistance genes were tracked in rice varieties based on breeding pedigrees, allele studies, and reactions to laboratory-reared planthopper colonies known as ‘biotypes’ (discussed in Section 3.3.4), and the varieties were deployed throughout Asia, often with detailed export and farmer adoption records [131]; (2) the SSST was sufficiently robust and simple, without the need for specialized equipment, to monitor virulence development under a diversity of conditions. Furthermore, through collaboration between IRRI and researchers throughout Asia, collections of breeding materials known as BPH nurseries (IRBPHN) and WBPH nurseries (IRWBPHN) were regularly dispatched for field testing under local conditions as part of the international rice testing program (IRTP) and later the international network for genetic evaluation of rice (INGER). Visible patches of ‘hopperburn’ also drew attention to early pockets of virulence adaptation in farmers’ fields and experimental plots [55,70,132,133,134]. (3) Resistance breeding was governed by planthopper responses to a set of differential rice varieties with identified or putative resistance genes. With the intention of determining the next gene for sequential deployment, researchers assigned local planthopper populations to specific ‘biotypes’ using these differential varieties and thereby monitored shifts in virulence against the associated resistance genes [135]. The events depicted in Figure 1 are summarized in the following paragraphs.

The first resistant HYVs released by IRRI included the varieties IR26, IR30, and IR46 that contained the *Bph1* gene (derived from Mudgo). These were widely promoted and adopted throughout the Philippines and Indonesia during the 1970s in response to widescale planthopper outbreaks [95,131]. Varieties with the *Bph1* gene were also planted in the Solomon Islands in the early 1970s, where rice production was largely confined to ca 1200 ha [111,169,170]. Furthermore, breeding materials with the *Bph1* gene were distributed in other countries, including Thailand, Vietnam, and Malaysia, where they were incorporated into national resistance breeding programs [95]. During the 1980s, *Bph1* was incorporated into Korean, Japanese, Taiwanese, and Chinese hybrid and *japonica* rice breeding programs, resulting in the release of several resistant, local varieties [56,109,173,186,188,189].

Rice varieties with the *bph2* gene (derived from ASD7) were first released by IRRI in 1976, of which IR36 and IR42 became popular in Southeast Asia [95]. Growing concerns about virulence adaptation to the *Bph1* gene in Southeast Asia meant that the *bph2* gene was often simultaneously incorporated with *Bph1* into breeding programs in Japan and China. Consequentially, hybrid varieties with *bph2* resistance were deployed in some parts of China before varieties with *Bph1* resistance [173]. Varieties with *bph2* were not widely adopted in the Solomon Islands because the deployed materials were coincidentally susceptible to leafrollers (*Susumia exigua* (Butler)) [169,170].

Finally, several rice varieties (e.g., IR56, IR62) and breeding lines with *Bph3* resistance were released by IRRI beginning in the early 1980s. By this time, varieties with PTB33 as a resistance donor, and containing the *Bph3* gene locus (likely containing the *Bph32* gene [190]), had already been deployed by breeding programs in South Asia (i.e., BG 379-2 [89]). Certain varieties with *Bph3* (e.g., IR62) were promoted in areas (Vietnam and Mindanao) with a high incidence of tungro disease because of their resistance to leafhoppers [106,191]; however, *Bph3* resistance never attained more than 10% of the planted rice area in the Philippines or 1% in Southeast Asia [99]. The variety Rathu Heenati was used as a differential variety to track virulence against *Bph3* [113,114,116,140,143], although accessions of Rathu Heenati are now known to also contain other resistance genes (i.e., *Bph14* and *Bph17* [192,193,194]) and some varieties believed to contain the *Bph3* locus may actually derive their resistance from the *Bph32* gene [195]. Only one IRRI variety (IR66) is purported to contain the *bph4* gene that is allelic with *Bph3* [99,194], and the gene was never widely deployed in Asia; nevertheless, monitoring for virulence against *bph4* was conducted at several sites using the differential variety Babawee [113,114,116,140,143].

The patterns of BPH virulence adaptation depicted in Figure 1 reveal the following features. (1) Virulence against the *Bph1* and *bph2* genes appear to pre-date the deployment of resistant HYVs in much of South Asia. Evidence suggests that some South Asian BPH populations were also virulent against varieties with *Bph3* and *bph4* before corresponding varieties were ever released by IRRI or other breeding programs [65,89,137,143,196]. This is probably because the genes originated from the region (i.e., Mudgo and ASD7 from India, Rathu Heenati and Babawee from Sri Lanka), but were first identified using East Asian planthopper populations. (2) Virulence adaptation in Southeast Asia occurred despite the largescale, concurrent planting of susceptible rice varieties: For example, hopperburn occurred in fields of IR26 (*Bph1*) 1 to 3 years (<10 generations) after initial deployment in the Philippines, Indonesia, and Vietnam [146,147,155,156,157,159,165,166]. In the same regions, virulence developed on *bph2* varieties within 2 to 6 years (<20 generations) after initial deployment [113,114,116,134,148,149,151,159]. At the time of widespread virulence against *Bph1*, only about 7% of rice planted in Indonesia included modern varieties with the *Bph1* gene [131]; by the time virulence against *bph2* resistance had become widespread, an estimated 60% of rice fields were planted with modern rice varieties that had the gene; this was largely due to the popularity of IR36 and Cisadane [45,131]. Virulence was often first noted in localized areas with intensive crop production practices, including excessive pesticide use [45,112,158]. Planthoppers are known to disperse widely and migrate over long distances such that adjacent susceptible varieties should have represented a considerable resistance refuge to slow the development of virulent populations (according to current theories of plant–herbivore co-evolution [49,197]). But this apparently did not occur.

Finally, (3) damage to rice varieties with *Bph1* and *bph2* genes in northern Vietnam, China, Korea, and Japan only began to occur in the late 1980s (*Bph1*) and early 1990s (*bph2*), with virulence increasing over the next decade [101,122,176,185,198]. Virulence likely first developed in northern Vietnam or southern China about 10 years after *Bph1* resistance was widely deployed in the region. This slower adaptation rate (compared to tropical regions) may be due to lower temperatures and the absence of overwintering BPH populations in these regions, less intensive rice production at the time, or the frequent depletion of virulence due to northward migrations and low winter survival rates [41].

#### 3.3.2. Relict Populations

**Figure 2:**
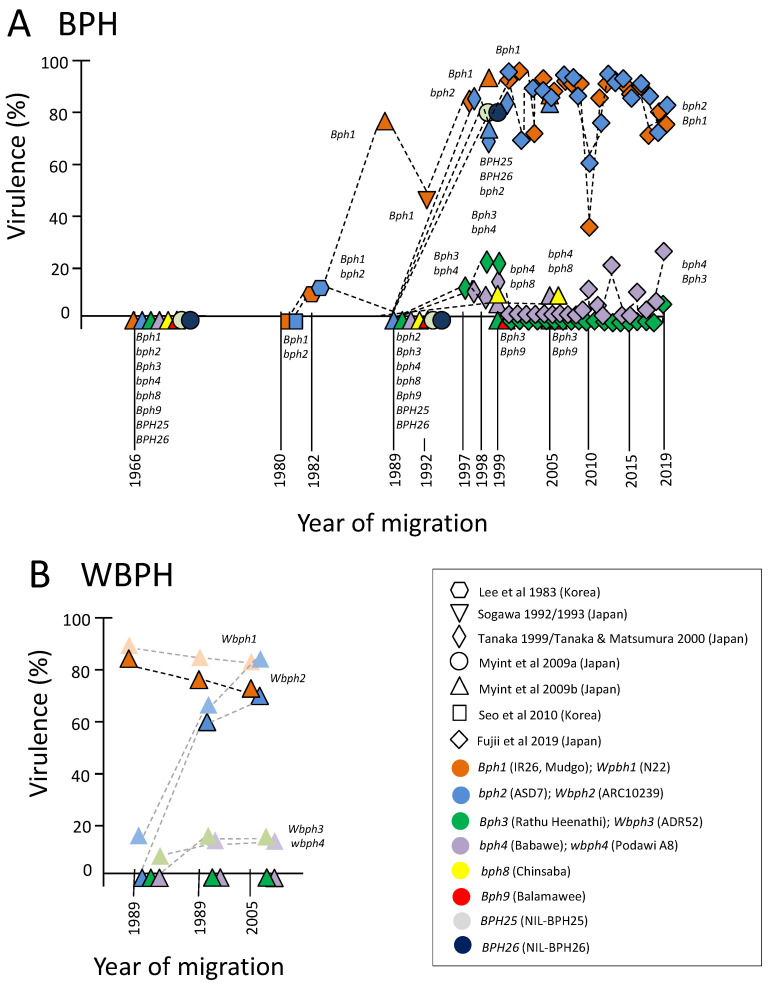
Estimated occurrence of virulence against specific resistance genes among migratory (**A**) brown planthopper (BPH) and (**B**) whitebacked planthopper (WBPH) populations arriving to Northeast Asia (Korea and Japan) between 1966 and 2019. Estimates are based on virulence bioassays (swollen abdomens [200] or honeydew production [182,198]) with recently collected [105,182,185,198] or relict [101,104,119,126,199] planthopper colonies. Estimates are based on the assumption that populations maintained as laboratory colonies do not lose virulence when reared for several generations on a susceptible variety. Symbols represent information sources as indicated in the legend. Lightly shaded symbols in (**B**) indicate the results from adult survival tests; corresponding darker symbols are from swollen abdomen tests [101]. Genes indicated below the *x*-axis in (**A**) remained effective during tests in the corresponding years. Further details of each study are presented in Table S2.

Researchers in Japan and Korea have collected and maintained colonies of planthoppers (BPH and WBPH), which were initiated with migratory individuals that settled in rice fields at different sites in different years. Many of these colonies were maintained under controlled conditions, in closed colonies, over several years [101,104,125,126,199]. Despite rearing the colonies on susceptible rice varieties, they maintain their original virulence against resistance genes. Researchers have assessed the virulence of the colonies by testing individuals on standard differential rice varieties using honeydew or swollen abdomen bioassays [149,151,198,200]. This has revealed shifts over the years in the proportions of each population showing virulence against different genes. Figure 2 presents the main results from these studies for BPH (Figure 2A) and WBPH (Figure 2B).

As indicated in Figure 2A, the virulence of migratory BPH against the *Bph1* and *bph2* genes increased steadily since the early 1980s. From the mid-1990s, over 75% of migratory BPH were virulent against *Bph1* and *bph2* (with an apparent and unexplained decline only in 2010). Observations of BPH outbreaks in Japanese fields of Saikai 184 (*Bph1*) during 1990 confirm that virulence against the gene increased dramatically at about that time [182] and that the relict colonies reflected these changes despite being maintained for years in closed cages. Although individuals were generally tested for their virulence to only one gene (but see Sogawa (1993) [198]), the high levels of virulence against *Bph1* and *bph2* indicate that individual planthoppers are predominantly virulent against both genes (see also Naeemullah et al., 2009) [125]. Furthermore, shifts in virulence against *BPH25* and *BPH26* suggest that these genes are allelic with *Bph1* and *bph2,* or likely represent the same genes [97,100]. In contrast, virulence against *Bph3*, *bph4*, and *bph8* has remained relatively uncommon (5–20%). The tendency for high levels of virulence against *Bph3* and/or *bph4* to coincide with the relatively low virulence against *Bph1* and *bph2* (i.e., virulence against *Bph1* or *bph2* + virulence against *Bph3* or *bph4* and possibly *bph8* = ca 100%) suggests that there may be constraints to individuals having cross-virulence against these two groups of genes (i.e., counter-virulence) (but see Cheng 1985 [109]). The exposure of individuals to multiple resistance genes using, for example, successive honeydew bioassays is required to test this hypothesis. A recent report by Tabata et al. (2021) [201] suggests that migrant BPH arriving to Japan sequentially adapted over time to a series of resistance QTLs in the variety PTB33, resulting in a gradual increase in virulence against backcrossed monogenic (each possessing a single QTL) segregated lines homozygous for PTB33 or T65. In a related study by Myint et al. (2009) [101], WBPH females with virulence against *Wbph1* were already common in a population collected in 1989 and remained high in two further collections (1999 and 2005) (Figure 2B). Furthermore, virulence against *Wbph2* dramatically increased to over 60% between 1989 and 1999. Meanwhile, virulence against *Wbph3* and *wbph4* appears to have remained low until 2005, when individuals were last collected.

#### 3.3.3. Selection Studies

The nature and rates of adaptation by planthopper and leafhopper populations to resistant rice varieties have been investigated in a number of studies that restricted populations to resistant varieties and monitored virulence periodically during the course of selection. Details of these studies are presented in Table S3. At least 17 BPH selection experiments have been reported [35,36,37,91,92,102,108,109,119,151,171,202,203,204,205,206,207,208] and one was reported for WBPH [209] (Table S3) (these do not include post-selection studies or selection studies related to adaptation mechanisms—see below). These studies reported gradual, proportional improvements in feeding efficiency (as determined by choice experiments, honeydew excretion, electro-penetration graphs (EPGs) or feeding marks) and survival or development (nymph survival, survival to adult, development time, adult longevity, nymph weigh, adult weight, weight gain during 24 h, wing dimorphism, or sex ratios). Adaptation to feed on monogenic resistant varieties generally occurred in less than 15 generations. Only seven of the studies [36,37,102,202,205,207,209] included aspects of reproductive success (pre-oviposition period, copulation rate, egg-laying/fecundity, egg survival/hatchability, population growth rates) in their monitoring of selection. These have been the most informative because the ability to oviposit on resistant rice appears to represent a greater barrier to adaptation than the ability of nymphs to successfully feed, survive, or develop on the varieties. For example, Ferrater et al. (2015) [37] found that oviposition rates of BPH reared for 20 generations on IR62 or PTB33, although improved over time, still remained below rates observed on the susceptible variety TN1. Furthermore, Li et al. (2014) [207] noted that, whereas fitness parameters in nymphs either improved rapidly or were not different when reared on the susceptible Minghui 63 and a near-isogenic line (NIL) with the Minghui 63 background and the *Bph15* gene locus from the wild rice *Oryza officinalis*, egg-laying and hatchability had not significantly improved after seven generations of selection. In studies that compared adaptation rates, planthoppers adapted more quickly on monogenic resistant varieties than on varieties with more than one major gene [37,92].

Six studies have examined virulence adaptation in GLH [24,93,106,118,127,129,210] and one examined virulence adaptation in the green rice leafhopper (GRL) [211,212] (Table S3). These studies were consistent in indicating a rapid improvement (within two to five generations) in the feeding success, survival, and development of nymphs on resistant plants. However, the rates of improvement across parameters often varied considerably, and improvements in oviposition rates were relatively slow. For example, in a study by Vu et al. (2014) [93], GLH nymphs had adapted to survive, feed, and gain weight on a *Grl2*+*Grl4* pyramided line (PYL) within five generations, but had not significantly improved in their ability to oviposit on the plants even after 10 generations (see also Heinrichs and Rapusas 1990 [118]). As with planthoppers, rates of adaptation appear to vary depending on the variety exposed, and also between different studies that use the same varieties (i.e., selection on Pankhari 203 took three generations in a study by Takita and Habibuddin (1985) [127], but nine generations in a study by Heinrichs and Rapusas (1990) [118]). Unfortunately, a majority of studies that compared leafhopper selection on a range of resistant varieties (including varieties with one or multiple genes) did not include replicates in their designs (Table S3).

#### 3.3.4. Post-Selection Populations and the Utility of ‘Biotypes’

**Figure 3:**
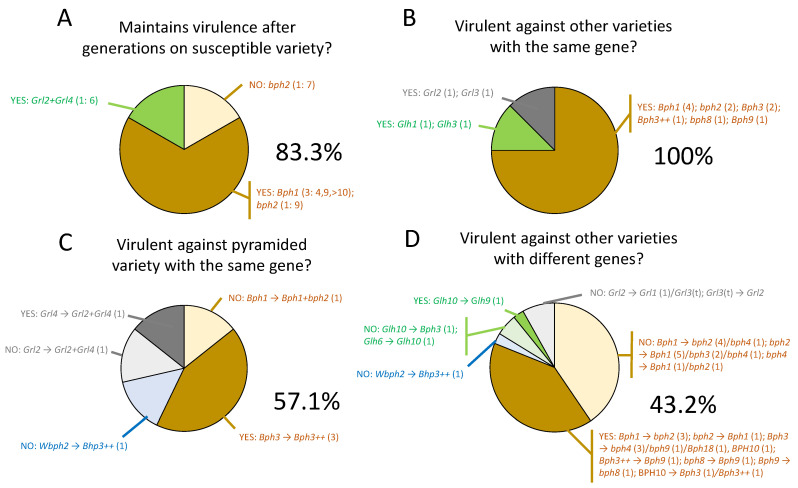
Summary of results from virulence tests with adapted planthopper and leafhopper populations. Results are presented as (**A**) virulence after generations on a susceptible variety, (**B**) virulence against monogenic varieties with the same genes as the natal host, (**C**) virulence on pyramided lines/varieties with the same gene as the natal host, and (**D**) virulence on varieties with different genes to those of the natal host. Genes of natal hosts are indicated in (**A**) with the number of tests and generations in parentheses (e.g., 1:7 = 1 test with 7 generations on the susceptible variety); genes of natal hosts are indicated in (**B**) with the number of tests in parentheses; genes of natal hosts are indicated in (**C**) with genes of pyramided lines/varieties and number of tests in parentheses; genes of natal hosts are indicated in (**D**) with genes of exposed varieties and number of tests in parentheses; percentages indicate the percentages of positive results based on accumulated tests. Further details of each study are presented in Table S4 [36,91,99,102,103,106,108,109,124,129,171,202,203,205,209,211,213,214,215,216,217].

Figure 3 summarizes the main results from experiments that exposed rice varieties to selected planthopper and leafhopper colonies adapted to one or more genes. These experiments have indicated four major features of virulence adaptation. (1) Virulent planthoppers and leafhoppers often continue to show preferences for susceptible varieties in choice settling and oviposition experiments [106,109,124,211]. However, even after rearing on susceptible varieties for several generations, they still maintain their original virulence [91,124] (Figure 3A). This is further corroborated from tests with relict populations [101,105] (see Section 3.3.2). (2) Planthoppers and leafhoppers adapted to one monogenetic resistant variety can successfully feed, survive, develop, and reproduce on other monogenetic resistant varieties that possess the same resistance gene [36,103,109,204,205,212,213]; however, studies have also shown that the levels of virulence, as indicated by comparative fitness tests on similar monogenetic varieties, will often vary between varieties [99,108], possibly due to undetermined resistance QTLs, but also due to ‘feeding inexperience’ and the genetic distance between varieties (Figure 3B). (3) Planthoppers and leafhoppers adapted to monogenic resistant varieties can overcome resistance in lines with the same gene together with other different genes (i.e., two or more genes in a single variety). This is suggested from only four studies, three of which challenged PTB33 and/or Rathu Heenati with *Bph3*-adapted BPH populations [99,103,214] (Figure 3C). Finally, (4) planthoppers and leafhoppers selected for virulence to monogenetic resistant varieties with one gene, will sometimes express virulence against varieties with other, different genes [91,99,102,103,106,109,171,203,209,214] (Figure 3D).

Laboratory-selected planthopper and leafhopper colonies have been useful to predict responses to varying levels and sources of rice resistance (Figure 3); however, the utility and predictive ability of such inbred colonies, often referred to as ‘biotypes’, is limited. As discussed in Section 3.3.1, biotypical responses to a small number of known resistance genes were useful in tracking the predominance of adaptations to early deployed resistance mainly based on IRRI breeding materials (Figure 1). However, the biotype concept has often been applied without a clear understanding of what a ‘biotype’ actually constitutes or without recognizing the limitations of the concept for research on virulence. For example, the term biotype has been used to describe selected, highly inbred laboratory colonies that show predictable responses to resistant varieties or resistance genes [33,127,136,196,202,211,212,218,219,220,221]. Such colonies can be derived from a single female [121,222], thereby indicating that the term biotype could be applied to any individual with a defined suite of heritable traits. The term biotype has also mistakenly been assigned to cryptic species, such as *Nilaparvata muiri* China, which feeds on *Leersia* spp. [221,223,224,225,226,227,228,229]. The term is also applied to represent wild populations that have significant proportions of individuals adapted to specific resistance genes. For example, several recent studies have proposed genes that function against specific numbered biotypes [90,162,230,231,232], and breeding and deployment programs are frequently guided by the idea that some field populations are biotypes that can be managed by sequentially releasing novel resistance genes [63,135,137,140,147,162,171,221,233]. Practical issues around the naming of biotypes have also emerged and, as the number of recognized resistance genes has increased, the naming of biotypes using sequential numbers has become untenable [90,162,163].

Further confusion has resulted from pseudo-replicated studies, without adequate experimental controls, which differentiated inbred colonies based on anatomical, physiological, or behavioral traits [34,216,227,234,235,236,237,238,239,240]—similar to several recent studies that analyzed divergence in planthopper microbiomes on unreplicated inbred colonies (see below). Subsequently, this led to a proliferation of studies that attempted to identify biotypes from field collections using population-level genetic markers [227,241,242,243,244,245,246]. It is now clear that virulence is a heritable trait, and that at population levels, it is influenced by several environmental variables, including the prominence of deployed resistance in the environment [49,150,247]. Therefore, populations will include individuals that differ in their virulence to a range of different genes, with shifting proportions of distinctly virulent individuals that are otherwise similar across a majority of other traits [241,248,249,250,251,252,253]. It is therefore inadvisable to base breeding and deployment strategies on laboratory biotypes without more detailed studies of virulence representation (i.e., proportions of individuals with virulence against many different genes) in target populations. This is particularly relevant to modern research that has integrated larger numbers of resistance genes and alleles into breeding programs (compared to the two to four genes used in the 1970s–1990s) and because many programs now focus on pyramiding multiple resistance genes into selected, high-yielding rice varieties (see Section 3.5.1). Because of its frequent misuse, references to biotypes have been largely replaced by more careful descriptions of populations that better represent variations in time (e.g., years) and space (e.g., collection sites) [101,105,250]. A number of review and opinion papers provide further discussions on the utility and limitations of the biotype concept [120,135,221,254,255,256,257].

### 3.4. Mechanisms of Virulence Adaptation

Two aspects of the interactions between planthoppers or leafhoppers and resistant rice varieties should be considered when elucidating the mechanisms of virulence adaptation. Firstly, events during different stages of herbivore attacks and herbivore responses to plant defenses can be distinguished as (a) normal responses to susceptible varieties (i.e., particularly during the first ≤ 48 h, including what is sometimes known as the assault stage) that are accompanied by detectable shifts in gene regulation and metabolic proteins; (b) responses to polygenic (i.e., influenced by two or more genes) but minor differences in susceptible and resistant varieties, particularly during the assault stage and in early generations of selection (often occurring during generations within a single rice cropping season), which may include shifts in endosymbionts and non-heritable (i.e., acclimation) or heritable improvements in herbivore responses to plant defenses, nutrients, and secondary metabolites (e.g., phenolic and oxalic acids, flavonoids, phytosterols, and polyamines [258,259,260,261,262,263]); and (c) population responses to major genes and their associated resistance mechanisms that may change quantitatively or qualitatively over time (i.e., generations) through adaptive selection [35,49,264,265]. The current literature is often vague in separating these three possibilities. Secondly, virulence adaptation should be regarded as a complex of responses that can be divided into several categories, including behavioral responses and molecular regulatory or metabolic responses; epigenetic changes in the herbivore or shifts in the genetics of herbivore populations related to feeding or food assimilation efficiency; epigenetic or genetic shifts in populations of key endosymbionts that alter their functional responses to rice defenses; and quantitative and qualitative changes in endosymbiont communities or in the herbivore microbiome [35,266,267,268]. Furthermore, these categories may respond to different aspects of the herbivore–plant interaction, including host finding, feeding, food digestion, nutrient assimilation, fecundity, hatchability, and others [247,268,269]. Categories of virulence responses and the evidence to support their roles during adaptation are discussed in this section.

#### 3.4.1. Virulence Responses to Novel Hosts

Planthoppers and leafhoppers exhibit changes in their behavioral responses to resistant varieties compared to susceptible varieties [46]. Among the best studied are herbivore feeding responses. Planthoppers and leafhoppers on resistant rice will generally probe more frequently and have shorter feeding bouts, as revealed during EPG studies [270,271,272]. Furthermore, planthoppers and leafhoppers excrete less honeydew on resistant rice and the honeydew often contains higher amounts of acidic wastes that are derived from xylem feeding and lower concentrations of sugars and other amino acids [37,271,273,274,275,276,277]. These observations suggest that the herbivores have difficulty in finding the phloem tubes; however, planthoppers and leafhoppers might also increase xylem feeding to dilute defensive secondary metabolites in the phloem of resistant rice, as occurs with aphids [37,278,279]. Furthermore, the honeydew composition may change over time to indicate that planthoppers, and possibly leafhoppers, improve their feeding efficiency within hours of the initiation of feeding [280]. The time for such improvements could be related to the relative susceptibilities of varieties or the genetic distance between host varieties. Planthoppers on resistant varieties may also change their feeding positions to avoid the plant’s defenses [281], and feeding by some species may be facilitated through prior feeding by virulent conspecifics or by other phloem-feeding species [280,282,283]. There is little evidence to suggest that such rapid improvements in feeding or in other responses to resistance are maintained across generations; however, the ability to rapidly improve feeding and other responses on resistant varieties likely plays a role during partial adaptation (i.e., where individuals have adapted to some aspects of resistance, but not all [93,118]).

A number of studies have assessed molecular responses by planthoppers on susceptible and resistant rice. These studies indicate variations in gene expression and related products in response to feeding on different rice hosts. Such responses typically occur in the first hours, during the assault stage [284,285,286,287,288], and may converge towards observed responses during comparative feeding on susceptible varieties after some hours [284]. Such plant-induced herbivore responses, which may differ between virulent and avirulent planthoppers, include changes in the expression of long, non-coding RNA and mRNA [289,290], changes in alternative splicing transcripts from regulatory to metabolic roles [291], the production of pancreatic triglyceride lipase [292] and other enzymes that inhibit plant defenses [293,294], the production of specific P450s (cytochrome P450 monooxygenases) involved in detoxifying defense chemicals [291,295], and changes in gene expression related to methylation during feeding [296,297]. It is difficult to determine whether such short-duration responses are associated with naivety during exposure to novel varieties, which could be susceptible or resistant, and therefore could be due to minor differences in host varieties or major resistance genes. Nevertheless, some evidence suggests that changes in the relative expression of certain P450s may be more frequent or permanent in adapted populations [295]. These might also be associated with epigenetic shifts towards adaptation [296]. For example, gradual improvements in feeding on susceptible varieties over two to three generations (i.e., the usual number of generations occurring during a single crop season) [37,91,92] could be due to epigenetic or other heritable but reversable shifts in response to minor, polygenic differences between rice varieties [267,296,297]. Further studies are required to investigate such possibilities, including studies that compare gene expression by partially and fully adapted planthoppers and leafhoppers in response to a greater variety of rice hosts, including different susceptible varieties.

#### 3.4.2. Virulence Adaptation during Sustained Exposure to Resistant Rice

**Figure 4:**
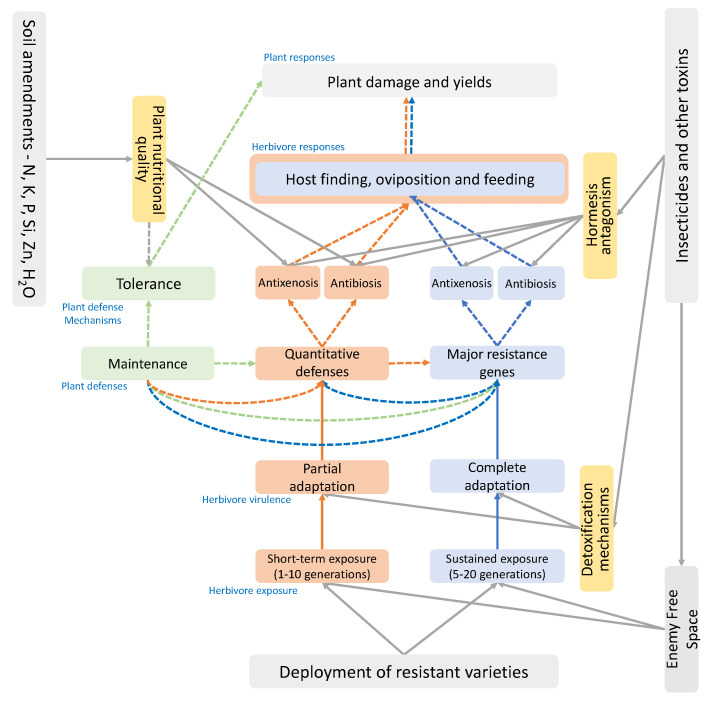
Model of virulence adaptation by planthoppers and leafhoppers to host varietal resistance. Solid arrows indicate interactions that promote virulence adaptation, dashed arrows indicate beneficial interactions in terms of crop yields. The model includes physiological adaptations to quantitative traits (factors indicated in orange) and evolutionary biological adaptations to major resistance genes (factors indicated in blue). The model differentiates partial adaptations to short term exposure (in orange boxes) that may be facilitated by environmental conditions (indicated in grey) such as soil nutrients that enhance the host’s nutritional quality and pesticides that enhance herbivore virulence directly (hormesis) or are otherwise antagonistic to the plants’ major defenses, and complete adaptations (in blue boxes) that respond to major resistance genes and may involve ‘gene-for-gene’ virulence genes that occur or evolve in rare forerunners and are selected during sustained exposure. Pesticide-induced enemy free space may accelerate such evolutionary adaptations, particularly if accompanied by hormesis, by reducing natural enemy-related trade-offs and allowing rapid population growth. Insecticides may further affect evolutionary adaptations by enhancing detoxification mechanisms in the herbivore or its symbionts. Note that tolerance (in green boxes) reduces herbivore damage and functions in synergy with resistance but exerts no selection pressure on herbivores.

A growing number of studies have investigated the potential mechanism underlying virulence adaptation to rice with major resistance genes during selection experiments that encompass multiple (≥five) herbivore generations. These studies and their main findings are listed in Table S5. Peng et al. (2017) [270] showed that when feeding on an artificial diet with ethanol extracts of YHY15 (with the *Bph15* gene), BPH populations selected on YHY15 had a higher transcript level of CYP4C61 (one of several P450 encoding genes) than BPH populations selected on TN1; the CYP4C61 gene of virulent and avirulent BPH also differed by one amino acid. This corroborates evidence for the role of P450s in detoxifying the secondary metabolites that underlie rice resistance [291,295] and suggests that elevated levels of these enzymes, or variations in the compositions of P450s in virulent and avirulent planthoppers during feeding on resistant rice, are heritable responses to selection. Shifts in detoxification mechanisms among herbivore populations may also include responses to other environmental toxins, such as pesticides, that simultaneously increase herbivore virulence against host plant defenses [298]. Such shifts can be epigenetic in nature. For example, the frequency of epigenetic modifications, including DNA methylation, changes during the selection of BPH on Rathu Heenati (*Bph3*/32) [267]; these changes are heritable, but are unstable after the selection pressure is removed. Epigenetic shifts in gene expression might, for example, explain why leafhoppers seem to ‘adapt’ rapidly (e.g., ≤five generations) to feed on resistant plants with adaptations that are relatively unstable, but adapt more slowly (≥ten generations) to lay eggs on the same resistant varieties [93,129]. In such cases, feeding might be related to multiple minor differences across varieties (polygenic resistance) and oviposition related to major resistance genes, with herbivore selection responses to the former mediated through epigenetic or other reversable physiological adaptations and to the latter through evolutionary biological adaptations [299] (Figure 4).

A number of recent reviews and opinion pieces have outlined some probable genetic determinants of virulence in BPH with a focus on potential gene-for-gene adaptation responses [247,268,269]. Early studies (pre-2000s) conducted reciprocal crosses and backcrosses of BPH populations selected on resistant and susceptible hosts to determine virulence inheritance patterns and the possible involvement of virulence genes. These mainly focused on virulence against *Bph1* and *bph2* resistance in Mudgo and ASD7, respectively. Evidence from these studies indicated that virulence was an inherited trait, but evidence varied as to whether this involved recessive or dominant genes, and whether virulence was sex-linked or not. Virulence against Mudgo (*Bph1*) has been attributed to a recessive or partially dominant gene [9,109,300]. More recently, Jing et al. (2014) [301], using high-density linkage mapping, associated the *Qhp7* locus (Chromosome 7) with BPH preference and the *Qgr5* and *Qgr14* loci (Chromosomes 5 and 14, respectively) with nymph growth on Mudgo (*Bph1*). Using BPH populations that were virulent against Saikai 190 (*Bph1*), Kobayashi et al. (2014) [302] determined that virulence (based on the quantities of honeydew excreted) was associated with a recessive *vBph1* gene (Chromosome 10). Virulence against ASD7 (*bph2*) and IR42 (*bph2*) has also been attributed to recessive or partially dominant virulence genes [148,300], and virulence against H105 (*bph2*) to a dominant or partially dominant gene [9,109]. Tanaka (1999) [222] found little evidence for a genetic correlation between virulence against Saikai 190 (*Bph1*) and ASD7 (*bph2*), thereby indicating that virulence to different genes and alleles was controlled by distinct genetic mechanisms. Whereas Sogawa and Kalin (1984) [148] indicated that virulence against IR42 (*bph2*) may be female-linked (see also Liu et al. (2005) [303]), Cheng (1985) [109] found no evidence for sex-linked virulence against Mudgo (*Bph1*) or H105 (*bph2*). However, these latter studies did not control for possible maternally transmitted, microbiome-related effects [121] (see below).

Despite identifying potential virulence genes and QTLs, there is still uncertainty about several issues. For example, based on the results of Jing et al. (2014) [301], it seems probable that component responses by virulent planthoppers to resistance are governed by different virulence genes, and these may include epigenetically or genetically inherited virulence traits. This implies that virulence adaptation involves multiple herbivore genes [35] because relative resistance is likely polygenic, with different plant traits governed by distinct genetic mechanisms affecting suites of planthopper and leafhopper responses [28,46,92,264]. However, as cautioned by Kobayashi (2016) [247], virulence as an evolutionary, biologically adaptive trait should be regarded as a threshold condition that is attained by individuals; therefore, tests for virulence should avoid continuously variable responses in favor of responses with clear bimodal distributions, such as the volumes of honeydew excreted or the state of the female abdomen (i.e., swollen or not-swollen). Furthermore, to date, all studies that have focused on the genetics of virulence have overlooked the possible contributions of the planthopper microbiome [266]. For example, because microbes, including endosymbionts and yeasts, are passed through the egg from mother to offspring, virulence can appear to be sex-linked irrespective of underlying genetic mechanisms [121]. Nevertheless, the identification of virulence genes is a major step forward in understanding adaptation, and strongly supports the idea that planthopper populations include forerunners that are pre-adapted to specific host resistance traits [49,102,247]. Furthermore, observations that several resistance sources are determined by different alleles of the same resistance genes [97] suggest that forerunners will exist for each state of the gene as a consequence of co-evolution. Virulence selection therefore might appear to progress rapidly because it only requires shifts in the dominance of individuals with corresponding virulence genes and is therefore primarily an ecological, population response without the need for contemporary evolutionary changes.

#### 3.4.3. Microbiome Responses during Sustained Exposure to Resistant Rice

Much recent attention has been directed toward understanding the role of symbionts during virulence adaptation. Planthoppers and leafhoppers have associated microbiomes that consist of a rich diversity of bacteria, fungi, and other microbes [210,304,305,306]. Research has focused on bacteria and yeast-like symbionts (YLS) as the two main components of the planthopper and leafhopper microbiome that are potentially involved in ‘adaptation’. The probable role for microbes is linked to the occurrence of bacteria-like organisms in the stylets of planthoppers and the sieve tubes of rice during feeding events [307,308]. *Asaia* sp. bacteria have also been found in planthopper eggs and in egg-infested rice plants [309]. Furthermore, microbes in the saliva [307] and even in excreted honeydew [274,310] have been implicated as possible elicitors [311,312,313,314] that induce rice defense responses to planthopper and leafhopper attacks or, alternatively, that shift the plant’s defense responses from primarily herbivore-induced to pathogen-induced defenses during the assault phase [282]. For example, in a recent study, avirulent BPH were shown to gain a fitness advantage when feeding on a resistant rice host (IR62: *Bph3*/*32*) that was previously attacked by virulent planthoppers; furthermore, virulence was acquired by non-adapted BPH during interim feeding on a tolerant variety (Triveni), thereby suggesting that virulence was transmitted horizontally [282].

Using a variety of molecular techniques, several studies have compared bacterial communities associated with planthoppers that were reared over several generations on susceptible and resistant rice varieties [210,273,307,315,316,317,318,319]. In general, these studies have reported differences in the abundance of specific organisms and in the composition of the microbial community on the different varieties. Despite such accumulating evidence, research on the potential role for microbiota during virulence adaptation has been largely inconclusive because it has remained purely descriptive, lacks experimental manipulation, and continually fails to replicate treatments at the proper level (Table S5). Furthermore, because of the descriptive nature of the experiments, the results lack any clarity around cause and effect: for example, microbiome signatures associated with virulence might be responses to the host condition, mediated through feeding, and not necessarily determinants of feeding ability. Indeed, Deng et al. (2024) [319] found that the abundance of Ascomycete endosymbionts decreased over 5 days in response to artificial diets with methyl jasmonate, thereby indicating that some changes in the microbiome that occur in the insect host are secondary to virulence. Only one study has replicated virulent and avirulent colonies by initiating colonies using collected GLH individuals from different field sites [210]. Perhaps unsurprisingly, the composition of the microbiome was strongly influenced by the collection site, regardless of the rice host. Nevertheless, patterns in the abundance of six (*Candidatus sulcus* clade, *Dyella*, *Bosea*, *Mycobacterium*, *Sandaracinus*, *Dyadobacter*) bacteria were consistent with host virulence against rice lines with one or two resistance genes [210]. Changes in the abundance of these bacteria likely represent downstream responses to GLH adaptations for feeding on resistant rice that are nevertheless important for nutrient acquisition.

Yeast-like symbionts are essential for the normal development of planthoppers and are involved in nutrient cycling and the production of rare amino acids [208,320,321,322]. A recent review has indicated the many potential roles for YLS during virulence adaptation, including the provision of alternative sources of essential nutrients, detoxifying ingested substances including toxic secondary metabolites, or down-regulating plant defense genes [266]. Studies that examined the role of YLS in virulence adaptation have remained largely inconclusive for similar reasons as outlined above for microbiome studies. In the case of YLS, studies have mainly focused on shifting abundances or densities in response to sustained exposure to susceptible and resistant rice. These studies have shown that YLS initially decline in abundance during early generations of insect host exposure, before gradually increasing over subsequent generations [37,206,323]. However, such patterns have been inconsistent across susceptible and resistant hosts [37]; the improved capacities of planthoppers to acquire rare amino acids over the course of adaptation have been largely independent of YLS abundance [208]; and the relative fitness of planthoppers on susceptible and resistant hosts is maintained irrespective of whether the planthoppers are symbiotic or aposymbiotic (i.e., heat treated individuals with depleted YLS numbers) [214]. Furthermore, during reciprocal mating experiments, although virulence was associated with the female parent, there was only weak evidence for any effect of YLS density on the resulting virulence [121]. These results do not entirely refute hypotheses relating YLS to virulence because studies have not generally assessed possible shifts in the composition of the YLS communities, or the epigenetic or genetic make-up of the communities in regard to their possible functions during virulence adaptation. Lai et al. (2022) [323] have only recently detected proportional shifts in *Ascomycete*-like, *Pichia*-like and *Candida*-like yeasts in response to host resistance. As is the case with research on the genetics of virulence [247], future research on the potential role for the microbiome in virulence adaptation should more carefully link the microbiome to virulent planthopper or leafhopper individuals identified using threshold characteristics.

### 3.5. Deploying Resistance to Conserve Rare Genes

Planthopper and leafhopper adaptation to resistant rice varieties has occurred at different rates in response to the nature of resistance and the extent of exposure to the herbivores (Figure 1). Variability in adaptation rates indicates that these factors can be managed to preserve the utility of rare resistance genes and to prolong the durability of resistance.

#### 3.5.1. Gene Pyramiding and Durability

Researchers have mainly focused on managing the nature of resistance to prolong durability by pyramiding two or more major resistance genes to increase the strength of varietal resistance [32,41,49]. Furthermore, evidence indicates that the durability of major genes can be prolonged where resistance or tolerance is associated with quantitative traits [92,264,265,324]. Indeed, recent studies indicate that at least moderate resistance (either through quantitative resistance or major genes) is required for tolerance against BPH to be effective [325,326]. These ideas respond to the hypothesis that pyramiding resistance and tolerance loci/genes prolongs durability beyond that achieved through the sequential deployment of monogenic resistance by increasing the strength of resistance and slowing virulence adaptation (henceforth the pyramiding-durability hypothesis). The lasting resistance of traditional rice lines that have several major resistance genes, compared to monogenic lines, supports the idea that pyramided resistance increases durability [38,39]. Furthermore, selection experiments that compared virulence adaptation on monogenic varieties and on varieties with two or more major resistance genes suggest that multigene varieties are more durable [37,211]. However, such observations generally confound the durability related to major genes, quantitative resistance traits, domestic or wild-rice resistance sources, and herbivore naivety; furthermore, the numbers of different resistance genes in traditional varieties are often unknown and vary between accessions [100,194,195,249,327,328,329]. Therefore, improved tests of the pyramiding-durability hypothesis require better control of the genetics of resistance.

Despite considerable research attention into gene pyramiding and the development of a large number of research-, breeding-, and commercial-rice lines with two or more genes for resistance to planthoppers or leafhoppers, there is still no convincing study to indicate that pyramiding can prolong durability. For example, Horgan et al. (2018) [32] reviewed evaluations of 13 sets of NILs/PYLs that compared herbivore reactions to susceptible, monogenic, and pyramided lines with common backgrounds (the recurrent parents). A further ten publications have also compared BPH responses to NILs and PYLs [117,330,331,332,333,334,335,336,337,338]. All of the twenty-three sets were evaluated for comparative resistance strength, but only one of the sets was ever evaluated using comparative selection experiments for related durability, despite most of the papers claiming that durability was the main reason for pyramiding resistance. In the only paper that did examine durability, a PYL with *Grl2* and *Grl4* was highly resistant to GLH, but the related monogenic NILs were susceptible. Nevertheless, selection on the monogenic lines over multiple generations resulted in concurrent adaptation to the PYL [129]. Similarly, Tabata et al. (2021) [201] showed that BPH became sequentially adapted to resistance QTLs in PTB33, thereby reducing the effectiveness of resistance over time. This strongly suggests that planthoppers and leafhoppers will sequentially adapt to deployed genes that occur in both monogenic or pyramided varieties in the environment. Furthermore, the occurrence of planthoppers adapted to two or more resistance genes (Figure 2) suggests that forerunners with virulence against different combinations of major resistance genes occur in nature. For example, in a similar herbivore–cereal system, populations of the Russian wheat aphid, *Diuraphis noxia* (Kurdjumov), with virulence against multiple sources of wheat resistance, emerged in the USA during the early 2000s, thereby reducing the utility of practically all resistance sources at the time [339,340]. Therefore, pyramiding resistance could inadvertently accelerate virulence adaptation if deployment is poorly managed, by increasing selection for populations with a prominence of individuals that are virulent against a number of different genes. This would result in a faster adaptation by herbivore populations to sets of resistance genes than if the genes were deployed sequentially. Further research is required to better address the pyramiding-durability hypothesis.

#### 3.5.2. Varietal Deployment and Durability

The extent of resistance gene exposure to planthoppers and leafhoppers depends on the area over which varieties with resistance genes are deployed, as well as on the herbivore population pressure (i.e., the size of the herbivore population) [49]. Several climatic and agronomic factors affect planthopper population size; fertilizers and pesticides have received the most research attention [49]. The effects of these factors on planthopper populations and the consequences for adaptation have been reviewed by Horgan (2018) [49]. In brief, nitrogenous fertilizers reduce the resistance of rice to planthoppers and leafhoppers but increase rice tolerance [325,326,341]. Evidence from comparative studies of planthopper responses under varying soil nitrogen concentrations indicate that although resistance declines on both resistant and susceptible rice, the relative fitness of planthoppers on the resistant varieties remains comparatively low [49]. This suggests that nitrogen affects plant nutritional quality or other minor defenses [14] without affecting the impacts of the major resistance genes (Figure 4). Nevertheless, a higher fitness of planthoppers on resistant rice under high nitrogen exposure could potentially accelerate virulence adaptation by enhancing the relative reproductive success of virulent forerunners.

A number of studies have associated insecticides and other pesticides with a loss of anti-herbivore resistance in rice [16,17,18,45,49,112,138,158,170,342]. Evidence indicates that insecticides can increase the plant’s nutritional quality [49,343], thereby reducing resistance, but without affecting the relative resistance of susceptible and resistant varieties [49]. However, hormesis responses to resurgence pesticides allow planthoppers to overcome rice defenses, in some cases making resistant and susceptible rice equally attractive to ovipositing females for a period after the plants have been sprayed [343,344,345,346,347]. Such observations suggest that the functioning of major resistance genes is largely maintained despite the immediate effects of the pesticides. However, the repeated exposure of herbivores to pesticides in the environment can also affect virulence adaptation by providing sustained selection pressures that promote the emergence of detoxification mechanisms such as the epigenetic or genetically enhanced production of P450s [103,348,349,350]. A number of studies have found associations between resistance to insecticides and virulence against plant defenses among planthopper populations to support this hypothesis [103,272,351,352] (but see Fujii et al. (2024) [353]), often without clear knowledge of the underlying mechanisms (but see Pang et al. (2024) [298]). Finally, insecticides may increase the rate of virulence adaptation by providing enemy free space for virulent planthoppers, thereby further increasing the reproductive success and population growth rates of virulent forerunners (Figure 4) [49]. Heavy insecticide use probably accelerated virulence against *Bph1* and *bph2* in Indonesia during the 1970s–1980s, particularly since large tracts of traditional and improved varieties without resistance often dominated rice production landscapes at the time [131]. The avoidance of insecticides and the promotion of planthopper and leafhopper regulation by natural enemies are predicted to increase the durability of resistant rice [49].

The extent of varietal deployment has clearly affected virulence adaptation as is evident from the predominance of *Bph1*- and *bph2*-adapted BPH throughout Asia (Figure 1 and Figure 2). As indicated by Sogawa and Kalin (1987) [151], the proportions of individuals with virulence in sampled populations appear to reflect the extent of resistance deployment in the landscape. To reduce selection pressures, the deployment of any one gene should be restricted spatially and temporally. Spatially, gene deployment may be restricted by ensuring that susceptible refuges or varieties with different resistance genes (i.e., multilines) are deployed together [91,354,355,356,357]. Seed mixtures with relatively high proportions of resistant seed seem to be necessary to maintain the benefits of resistance, based on limited field trials [356,358]. Multiline landscapes have not been tested for their effects on virulence adaptation, although a diversity of resistance sources used in the Mekong Delta of Vietnam seems to have resulted in BPH populations with simultaneous virulence against several different genes [43,57,115]. Evidence from simulation experiments with BPH in cages suggests that multilines can delay virulence adaptation relative to cages with a single resistant variety. However, multilines appeared incapable of prolonging resistance beyond that of sequential deployment in the simulations [109,203]. The same result was found with GRH in cages with a single resistant rice line or mixtures of resistant rice lines [359]. The regional deployment of resistance genes that restricts certain genes to regions where planthoppers only occur as migratory populations has been proposed as a possible method to increase resistance durability [41]. This ‘migration-based deployment’ suggests that because planthoppers cannot overwinter in north temperate regions, and because return migrations are relatively small and occur when rice is less available in tropical fields, then virulent individuals fail to build up numbers when exposed to resistance in temperate regions despite continuous waves of migrants [41].

To reduce exposure temporally, resistance genes can be rotated by season or years. Simulation experiments using caged planthopper populations have suggested that rotations will prolong resistance [109,360]. Such rotated deployment may reduce the incidence of epigenetic virulence in populations [267] and reduce partial adaptations [107,118], thereby prolonging overall durability. Despite such positive results, in practical terms, it is difficult to ensure that resistance genes are rotated in the environment, particularly because farmers will often store seed for planting and because planthoppers and leafhoppers can migrate over long distances [41]. Further research is required to test these hypotheses and devise workable implementation schemes. Technologies that monitor virulence and resistance gene deployment, together with improved information sharing at regional and international levels, could improve deployment to counter evolutionary biological adaptations.

## 4. Conclusions

Although there are many sources of resistance (i.e., traditional varieties, landraces, and wild rice) against planthoppers and leafhoppers, these represent a relatively small number of major resistance genes. Therefore, strategies are needed to conserve resistance genes as public goods. Apart from *Bph1*, *bph2*, *Wbph1*, and *Wbph2*, there is no evidence of widescale planthopper or leafhopper adaptation to other major genes. Localized populations adapted to *Bph3*, *bph4*, and other genes have been reported. Reports of hypervirulent BPH populations in South Asia are probably due to the South Asian origins of many of the most effective resistance sources and, consequently, a predictably higher frequency of virulent individuals in the region. Virulence adaptation against *Bph1* and *bph2* occurred despite large areas of susceptible rice that should have reduced adaptation rates. Some pockets of virulence apparently occurred in pesticide-induced enemy free space under the intense rice production systems where resistant varieties were first deployed.

Simulation experiments have shown that adaptation to one major gene can reduce the utility of varieties, including pyramided lines, with the same gene, and sometimes reduces the effectiveness of varieties with other, different major resistance genes. Virulence is a complex trait: evidence suggests that adaptation may be partial or complete and related to detoxification and other mechanisms that are epigenetically or genetically inherited by the herbivores and/or possibly by their symbionts. Currently, there is little direct evidence for the role of symbionts in virulence adaptation. This is largely because of widespread pseudo-replication in relevant research that is probably a legacy of biotype studies and the confusion that the term ‘biotype’ has caused. Furthermore, a majority of studies have assessed virulence as a characteristic of populations and not individuals, thereby obscuring possible threshold states that are linked to major virulence genes. Similarly, although gene pyramiding is proposed as a method to prolong resistance durability, no study has adequately tested this hypothesis and there have been only a few studies of the effectiveness of different varietal deployment strategies. Based on existing evidence, variety rotations and migration-based deployment are likely to be more effective than multilines in reducing rates of adaptation. Multilines, like gene pyramiding, might actually accelerate virulence adaptation if they are not well managed.

Several topics related to virulence adaptation in planthoppers and leafhoppers require considerably more research attention. These include the following: research into the effectiveness of pyramiding to prolong durability; possibilities to map currently deployed genes that occur, deliberately or not, in commercial varieties and thereby affect the strategic deployment of genes; possibilities of counter-virulence where individuals are incapable of possessing virulence against two or more major genes; possibilities for the practical implementation of rotations, migration-based deployment, or multilines; an elucidation of the possible roles for symbionts and the insect microbiome in virulence adaptation using manipulative experiments; and the effects of pesticides in driving shifts in population virulence at landscape and regional scales.

## Figures and Tables

**Table 1:** Percentages of screened rice accessions with resistance to planthoppers and leafhoppers from studies with wild and cultivated rice. The numbers of accessions screened are indicated in parentheses.

Herbivores Species ^1^	Origin of Herbivore Population	Origin of Rice Accessions ^2^			
		East Asia	South Asia	Global Cultivated ^3^	Wild Species
*Nilaparvata lugens* (Stål), brown planthopper [BPH] ^4^	East Asia	3.8% (13,917) [11,56,57,58]	-	0.2% (ca 26,000) [59]	68.2% (88) [56]
	South Asia	-	8.7% (17,194) [10,27,60,61,62,63]	7.7% (3880) [64,65,66]	64.8% (128) [67]
*Sogatella furcifera* (Horváth), whitebacked planthopper [WBPH] ^5^	East Asia	-	25.4% (71) [68]	0.8% (46,488) [25]	37.7% (228) [69]
	South Asia	-	24.8% (514) [26,61,62]	29.9% (67) [70]	56.6% (297) [71,72]
*Laodelphax striatellus* Fallén, small brown planthopper [SBPH] ^6^	East Asia	20.6% (180) [73,74]			
*Nephotettix virescens* (Distant), green leafhopper [GLH] ^7^	East Asia	-	-	2.2% (50,333) [75,76]	67.2% (826) [69,75,77]
	South Asia	-	37.5% (24) [61]	-	-
*Nephotettix cincticeps* Uhler, green rice leafhopper (GRL)	East Asia			34.8% (164) [78,79]	
*Nephotettix nigropictus* (Stål) ^7^	East Asia	-	-	-	95.3% (91) [80]
	South Asia	-	33.3% (24) [61]	-	-
*Maiestas dorsalis* Motschulsky, zig-zag leafhopper [ZLH] ^8^	East Asia	-	-	2.1% (3870) [25,81]	55.2% (208) [69]
*Tagosodes orizicolus* (Muir) ^9^	Colombia	-	-	59.9% (534) [82]	-

^1:^ Abbreviations are indicated in square brackets; Heinrichs (1985) [55] presents information on screening techniques for planthopper and leafhopper species including the blue leafhopper, *Empoascanara maculifrons*, and white leafhopper, *Cofana spectra*; ^2:^ numbers are percentages of screened germplasm with at least moderate resistance (i.e., standard evaluation system (SES) ≤ 5 [83]), number of screened accessions are indicated in parentheses; only studies without pre-selection of potentially resistant materials are included; studies that potentially screened the same germplasm using different planthopper or leafhopper populations have been excluded; ^3:^ ‘Global cultivated’ refers to large germplasm collections screened for resistance that include cultivated (modern varieties, traditional varieties and landraces) rice collected throughout the world; ^4:^ high levels of resistance (i.e., SES ≤ 3) occurred in <5% of accessions (see also Pathak 1971 [84]), even for wild rice species (based on cited references); ^5:^ high levels of resistance occurred in <10% of accessions (based on cited references); ^6:^ high levels of resistance occurred in <10% (based on cited references); further studies, not considered in the table, indicate that 371 varieties/landraces were screened for resistance to SBPH and rice black-streaked dwarf virus (RBSDV), of which 14% were resistant [85,86]; 318 varieties/landraces were screened for SBPH and southern rice black-streaked dwarf virus (SRBSDV), of which 0.6% were resistant [87]; ^7:^ high levels of resistance occurs in wild rice accessions at >50%; ^8:^ previously known as *Recilia dorsalis* (Muir); high levels of resistance occurred in <1% of accessions (based on cited references); ^9:^ high levels of resistance occurred in ca 20% of accessions [82].

## Data Availability

No new data were created; tabulated data from existing publications are included, together with source references, in the Supplementary Materials.

## Supplementary Materials

The following supporting information can be downloaded at: https://www.mdpi.com/article/10.3390/insects15090652/s1, Figure S1: Planthopper life cycle and some common indicators of host plant quality used during rice resistance evaluations, Figure S2: Results after screening varieties for resistance to the brown planthopper using a modified seedling seedbox test (MSST), Table S1: Results of screening studies to determine virulence of brown planthopper populations to differential rice varieties with known or putative resistance genes, Table S2: Details of relict brown planthopper and whitebacked planthopper colonies initiated with planthoppers that immigrated into Korea and Japan, Table S3: Details of selection experiments conducted with Asian brown planthopper, whitebacked planthopper, green leafhopper, and green rice leafhopper on resistant rice varieties, Table S4: Information on the nature of virulence adaptation in the brown planthopper, whitebacked planthopper, green leafhopper, and green rice leafhopper based on post-selection studies, Table S5: Advances in understanding the mechanisms of virulence adaptation in the brown planthopper and green leafhopper.
