# Supplementary material for: Virulence Adaptation by Rice Planthoppers and Leafhoppers to Resistance Genes and Loci: A Review
Source: Insects. 2024 Aug 29;15(9):652. doi: 10.3390/insects15090652 (PMC11432362; doi:10.3390/insects15090652)
Supplement: Supplementary file 1 [file insects-15-00652-s001.zip › insects-3174540-supplementary.pdf]

Supplementary Information

Planthopper Life Cycle and Host Quality Indicators

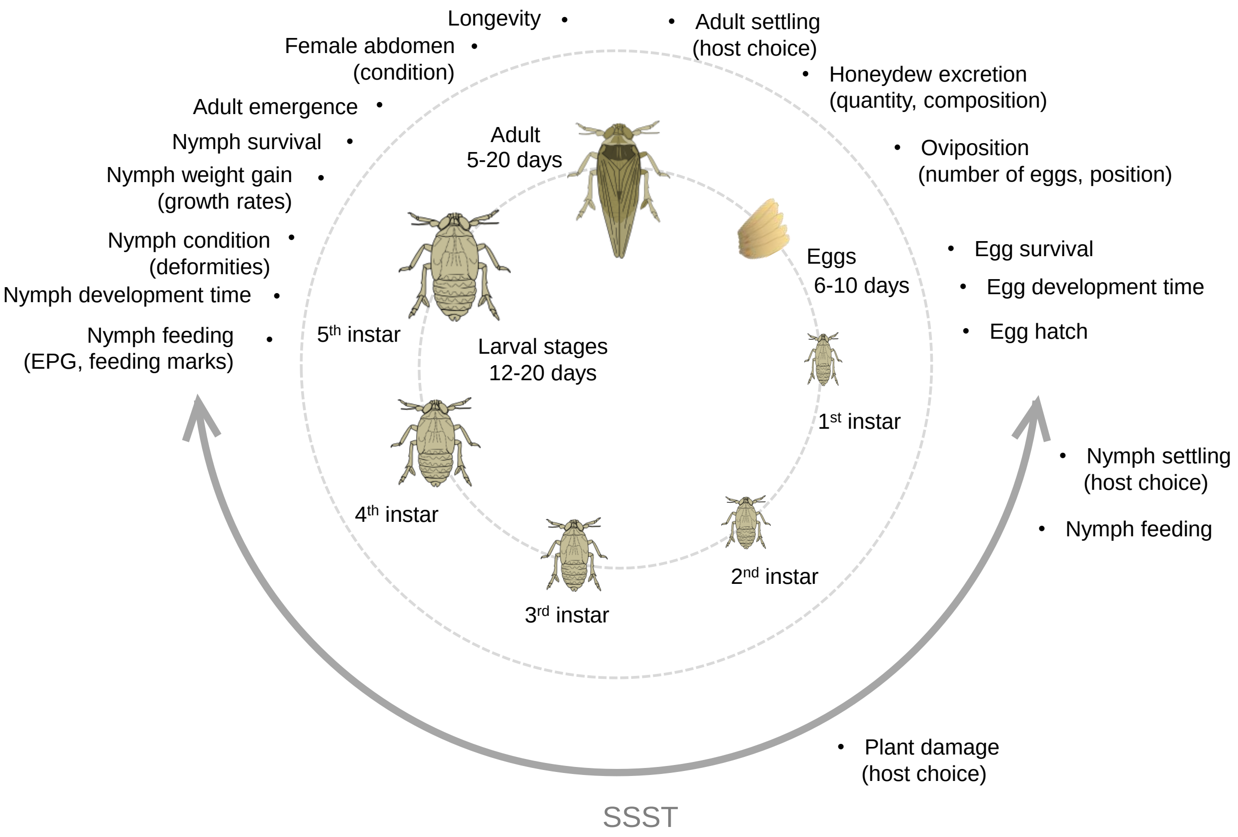

Figure S1. Planthopper life cycle and some common indicators of host plant quality used during rice resistance evaluations. The arrows indicate the scope of the standard seedling seedbox test (SSST)(see Figure S2), which is the most commonly used screening method to identify resistant rice varieties and wild rice species. Similar indicators and tests are applied during assessments of resistance against other rice planthoppers and leafhoppers. See Horgan (2009)[1] for further information on indicators and screening tests.

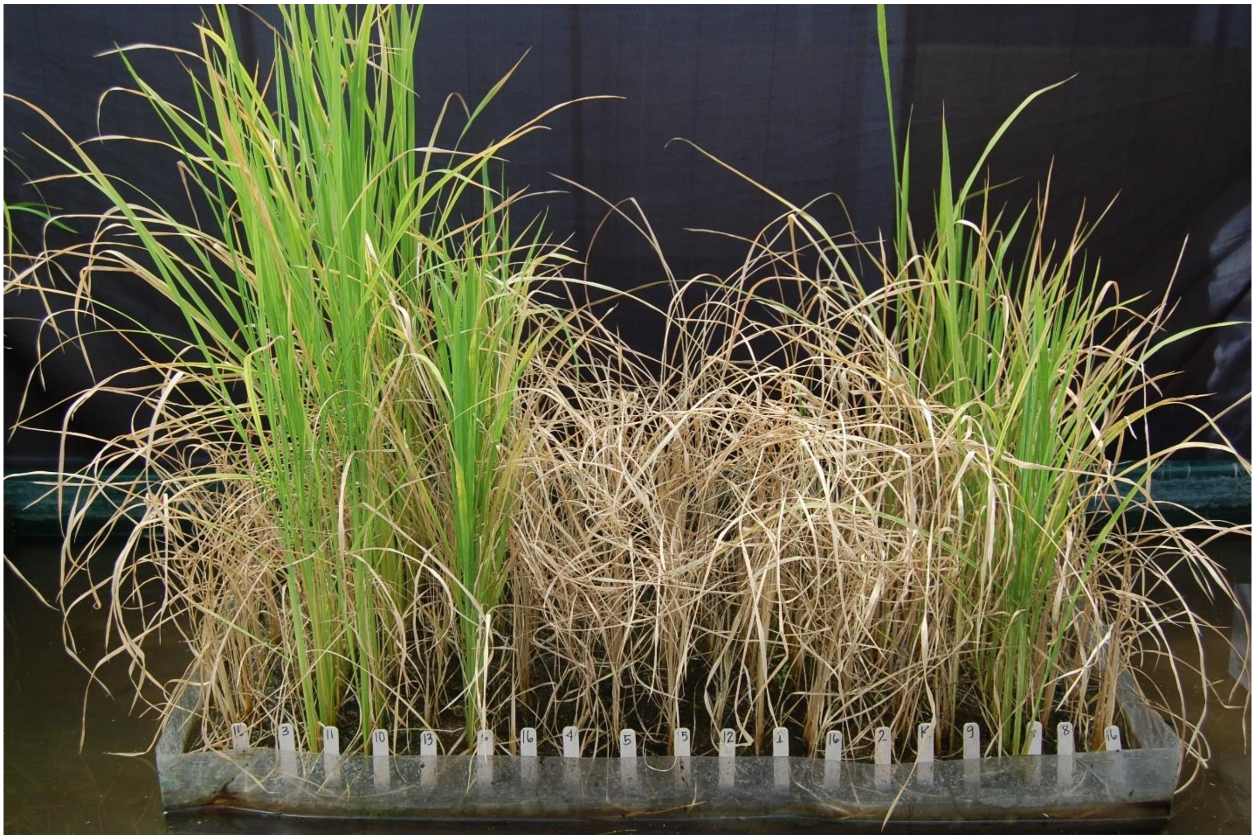

Figure S2. Results after screening varieties for resistance to the brown planthopper (*Nilaparvata lugens* (Stål)) using a modified seedling seedbox test (MSST). In the MSST, the seedbox is infested at a lower nymph density or at a later rice growth stage than the SSST, thereby, allowing nymphs to develop to adults and oviposit. See Horgan (2009)[1] and Horgan et al (2021)[2] for further details. Varieties: 1 = ASD7 (*bph2*), 2 = Asominori (ovicidal response), 3 = IR22, 4 = IR40 (*bph2*), 5 = IR46 (*Bph1*), 6 = IR62 (*Bph3/32*), 7 = IR65482-4-136-2-2 (*BPH10*), 8 = IR65482-7-216-1-2-B (*BPH18*), 9 = Mudgo (*Bph1*), 10 = PTB33 (*Bph3/32*, *bph2*, *Bph17-ptb*), 11 = Rathu Heenati (*Bph3/32*, *Bph 14*, *Bph17*), 12 = T65, 13 = TKM6 (*Bph1*), 14 = Triveni, 15 = Utri Rajapan, 16 = TN1; planthoppers were collected in Laguna, Philippines in 2010. Note resistance of lines 6, 7, 10 and 11. (Photo by Carmencita C. Bernal, International Rice Research Institute, the Philippines.)

Table S1. Source information for Figure 1. Results of screening studies to determine virulence of brown planthopper populations to differential rice varieties with known or putative resistance genes. Unless otherwise indicated, the results are based on the standard seedling seedbox test (SSST), or other similar preference tests, and evaluated using the standard evaluation system (SES). See Figure 1 for summarized information.

**Country**[1]	**Years of observation**[2]	***Bph1* (Mudgo)**[3]	***bph2* (ASD7)**[3]	***Bph3* (Rathu Heenati)**[3]	***bph4* (Babawee)**[3]
South Asia					
Bangladesh [3]	1981	S	S	R (PTB33)	
India [4]	1970s	S	S		
India (Pantnagar and Hyderabad)[5]	1976	S	S		
India (Pantnagar and Hyderabad) [6]	1979	S	S		
India (Tamil Nadu) [7]	1983		MS (damage in sprayed plots)		
India (Andra Pradesh) [8]	1983	S	S		
India [9]	1984		Moderate to heavy hopperburn by 1983		
India (Tamil Nadu and Pondicherry) [10]	1984	S	S	R	R
India (Cuttack) [11]	1985		S		
India (Tamil Nadu) [12]	1989	S	S	R	R
India (Raipur) [13]	1992	S	S	S	S
India (Raipur) [14]	1993	S	S	R	S
India (Maruteru) [15][5]	2004	S	R	S (IR62)	R
India (Hyderabad) [15][5]	2007	S	S	S (IR62)	S
India (Ludhiana) [15][5]	2007	R	R	R (IR62)	MR
India (Hyderabad) [15][5]	2010	S	S	S (IR62)	S
India (Telegana) [16]	2018	S	S	MR	S
Nepal (Parwanipur) [17]	1987	S	S	R	R
Sri Lanka [18]	1970s	S	S	S	R
Southeast Asia					
Indonesia (North Sumatra) [19]	1977	S	R		
Indonesia (North Sumatra, Banyuwangi and Bali) [20]	1978	S			

Indonesia (North Sumatra) [21]	1984		S		
Indonesia (North Sumatra) [22]	1984	S	S	R (IR56, Bahbolon)	
Indonesia (North Sumatra) [23]	1984		S		
Indonesia (Central Java) [24]	1987		R-MR (Cisadane losing effect)		
Indonesia [25]	1987		Deployed in 1980, no longer effective in North Sumatra by 1982 and in Central Sulawesi and Riau by 1983		
Indonesia (Subang) [15][5]	2011	S	S	S (IR62)	S
Indonesia [26]	2016		S		R
Indonesia Reviewed [27]	1970s-2000s	S	S		
Malaysia (East Peninsula) [28]	1986	S	S	R	
Malaysia (West Peninsula) [28]	1986	S	S	R	
Malaysia (Island) [29][4]	1989	R (13%)	R (20%)	R	R
Malaysia (Muda) [29][4]	1989	MR (40%)	MR (30%)	R (<10)	R (<10)
Malaysia [30][4]	1992	MR (34%)	S (92.2%)	R	R (9.6%)
Myanmar (Yezin) [15][5]	2013	S	S	R	S
Philippines (Mindanao) [31]	1975	S	R		
Philippines (south Luzon) [31]	1975	S			
Philippines (central Luzon) [32]	1976	S	R		
Philippines (Laguna) [33]	1978	S	R		
Philippines (Mindanao) [34]	1983		Hopperburn noted in sprayed plots		
Philippines (Mindanao) [35]	1985	S	S (after 7 years)	R (IR56, Rathu Heenati)	
Philippines (South Cotabato) [36]	1992			S (IR62)	
Philippines (Central Luzon) [37]	1996	MR (IR64)	MR (IR36)	MR (IR72)	
Philippines (Laguna) [15][5]	2009	S	S	MR	R
Philippines Mindanao) [15][5]	2012	S	S	MR	MR

Thailand [38]	1982	R			
Thailand [30]	1992	S (67.9%)	S (86.5%)	R	R
Thailand (Chainat) [39][6]	Early 2000s	0R/6	0R/6	5R/6	0R/6
Thailand (Phitsanulok) [39][6]	Early 2000s	5R/13	0R/13	12R/13	6R/10
Thailand [40][4]	2009	S	S	MR (28%)	S (87.5%)
Thailand [41]	2012	S		R	
Vietnam (Mekong Delta) [42]	1977	S	R		
Vietnam (Mekong Delta) [43]	1984	S	R		
Vietnam (Tien Giang, An Giang) [44]	1987	S	S	R	R
Vietnam (Angiang, Tiengiang, Haugiang, Minh hai, Cuulong) [45]	1990	S (except at Minh hai)	S	MS (except Minh hai)	MS
Vietnam (Haugiang, Angiang) [46]	1990	S	S (Angiang), MR (Huagiang)		
Vietnam (Hanoi, Tien Giang) [47]	1992	S	S (Tien Giang)	R	MS (Tien Giang)
Vietnam, Mekong Delta [30][4]	1992	S (ca 84%)	S (ca 84%)	S (ca 10%)	S (ca 13%)
Vietnam, Red River Delta [30][4]	1992	MR (ca 45%)	R (ca 20%)	R (ca 1%)	R (ca 1.5%)
Vietnam (Mekong Delta) [48]	1993	S	S	R	MS
Vietnam (Cuu Long) [49]	1997	MS	S	MR	MR
Vietnam (Ling Dinh) [15][5]	2012	S	S	MR (IR62)	S
Pacific Islands					
Solomon Islands [50]	1974	S			
Solomon Islands [51,52]	1985	Deployed 1974, ineffective by 1977		Deployed 1982, 60% hopperburn by 1984	
China and Northeast Asia					
China (Guangzhou) [53,54]	1984	R	R		
China (Hunan) [55]	1984	R	R		
China (Hunan) [56]	1987	R	MR		
China (tropical) [57]	1989	S	S		
China (Zhejiang) [57,58]	1989	S	R		
China (Guangdong) [59]	1991	MR	R	R (IR56)	
China (Hainan, Zhejiang, Guandong, Guangxi) [60]	1991	MR	R		

China (Guangxi) [58]	1992	S	S		
China (Hainan, Nanning, Fuyang) [61][4]	1993	MR	R	R	R
China (Guangdong) [59]	1995	S	MR		
China (Guangxi) [58]	1997	S	S	MS	
China (Yunnan) [58]	1997	S	S	MS	
China (tropical) [57]	1998	S	S		
China (Zhejiang) [57]	1998	S	S		
China (six provinces) [62]	2000s	S	S		
China (Zhejiang) [15][5]	2010	S	S	R	R
Taiwan (south) [63]	1971	R			
Taiwan [64]	1982		R		
Taiwan (Chiayi) [15][5]	2012	S	S	R	R
Japan (Kyushu) [65]	1992	MR	R		
Japan, Kyushu [30]4	1992	MR (ca 35%)	R (ca 10%)	R	R
Japan (Kumamoto) [61][4]	1993	R (<10%)	R	R	R
Japan [66][4]	1997	S (ca 70%)	S (ca 60%)	R (ca 15%)	R (ca 20%)
South Korea [67]	1978	R	R		
South Korea [68]	1980	R	R		
South Korea (Ashan, Hongseong, Boseong) [69][4]	1982	R (10%)	R (12%)		
South Korea [70]	1983	MS (damage to hybrid rice)	MS (damage to hybrid rice)		
South Korea [71]	1984	R			
South Korea [68]	1985-87	S (ca 60%)			
South Korea [68]	2005-07	S	S	R	

1: Countries and regions are indicated according to information given in the relevant publications; names, spelling and jurisdictions may have changed since the original publications. 2: Years of observation indicate the years in which field samples of planthopper populations were collected; in some cases where dates were not given, the year of publication is presented; 3: Genes are indicated with the main, associated differential varieties in parentheses; Mudgo, ASD7, Rathu Heenati and Babawee are traditional varieties that also possess other identified, putative and potential planthopper resistance genes; furthermore, the genetics of resistance associated with each differential may vary according to the specific accession used. It is assumed that susceptibility in these differentials/accessions shows adaptation to the indicated resistance genes; however, where the differentials remain resistant, it is not possible to determine from the data whether the planthoppers have adapted to the specified gene. S = susceptible, R = resistant, MS =

moderately susceptible (i.e., planthoppers gaining virulence, localized outbreaks observed), MR = moderately resistant (including possible tolerance). Parentheses indicate varieties used during screening if different from the differential variety. 4: Results are based on tests of individuals (e.g., honeydew tests) with results presented as percentages of tested individuals adapted to each differential variety. 5: MR indicates that the differentials were ranked highly (≤5) during SSSTs, but were not significantly different from the susceptible check TN1. 6: Numbers indicate the proportions of planthopper colonies from each site that were not adapted to the corresponding differentials (i.e., 6R/13 = differential appeared resistant to 6 of 13 test colonies tested).

Table S2. Details of relict brown planthopper (BPH) and whitebacked planthopper (WBPH) colonies initiated with planthoppers that immigrated into Korea and Japan. Differential varieties and bioassays used to assess shifts in virulence are indicated. See Figure 2 for further details.

**Species**	**Collection Sites**[1]	**Years**	**Colony Host**	**Differentials Used in Tests**[2]	**Tests**
BPH [69]	Korea - Southwest coast (Ashan, Hongseong, Boseong)	1982	Milyang23	Milyang23 (none); Cheongcheongbyeo (*Bph1*); Milyang63 (*bph2*)	SSST
BPH [65]	Japan - Kyushu	1990-1991	NA	Reihou (none), IR26 (*Bph1*), Saikai184 (*Bph1*), IR42 (*bph2*)	Honeydew test, oviposition
BPH [72]	Japan - Kyushu	1992	96 immigrant females	Reihou (none), IR26 (*Bph1*), Saikai184 (*Bph1*), IR42 (*bph2*)	Honeydew test (10mg = normal feeding)
BPH [66]	Japan - Isahaya-97; Isahaya-98; Isahaya-99; Katsumoto-98	1997-1999	Reiho, Shinrei, Mochiminori	Mudgo (*Bph1*), ASD7 (*bph2*), Norin PL 10 (*Bph3*), Babawee (*bph4*)	Swollen abdomen test [73]
BPH [74]	Japan - Nishigoshi-91	1991	Reiho	Mudgo (*Bph1*), IR26 (*Bph1*), ASD7 (*bph2*), Babawee (*bph4*), Saikai 190 (*Bph1*), Norin PL10 (*Bph3*)	Swollen abdomen test
BPH [75]	Japan - Hatano-66, Chikugo-89, Isahaya-99, Nishigoshi-05	1966, 1989, 1999, 2005	Reiho	T65 (none), *BPH25*-NIL, *BPH26*-NIL, *BPH25+26*-PYL, ADR52 (*BPH25+BPH26*)	Adult and nymphal survival, swollen abdomens, nymphal weight gain, oviposition
BPH [76]	Japan - Hatano-66, Chikugo-89, Isahaya-99, Nishigoshi-05	1966, 1989, 1999, 2005	Reiho	T65 (none), N22 (*Wbph1*), ARC10239 (*wbph2*), ADR52 (*Wbph3*), Podawi-A8 (*wbph4*), N'Diang Marie (*Wbph5*), Manggar (*Wbph5*), Mudgo (*Bph1*), ASD7 (*bph2*), Rathu Heenati (*Bph3*, *Bph17*), Babawee (*bph4*), Chin Saba (*bph8*), Balamawee (*Bph9*)	Swollen abdomen test
WBPH [76]	Japan - Chikugo-89, Nishigoshi-99, Nishigoshi-05	1989, 1999, 2005	Reiho	T65 (none), N22 (*Wbph1*), ARC10239 (*wbph2*), ADR52 (*Wbph3*), Podawi-A8 (*wbph4*), N'Diang Marie (*Wbph5*), Manggar (*Wbph5*), Mudgo (*Bph1*), ASD7 (*bph2*), Rathu Heenati (*Bph3*, *Bph17*), Babawee (*bph4*), Chin Saba (*bph8*), Balamawee (*Bph9*)	Swollen abdomen test
BPH [68]	Korea - S-BPH	1980s	Chucheongbyeo	Ilpumbyeo (none), Cheongcheongbyeo (*Bph1*), ASD7 (*bph2*), M63 (*bph2*)	Nymph survival, electrical penetration graphs (EPG)

BPH [77]2	Japan - Chikushino, Isahaya, Koshi, Minami-Satsuma	2001-2019 (except 2002)	mainly within 12 months; Reiho	2001-2005: TN1 (none), IR26 (*Bph1*), ASD7 (*bph2*), IR42 (*bph2*), Kanto-PL7/Norin-10 (*Bph3*), Babawee (*bph4*); 2006: TN1 (none), Mudgo (*Bph1*), IR42 (*bph2*), Kanto-PL7 (*Bph3*), Babawee (*bph4*); 2007-2019: T65 (none), Mudgo (*Bph1*), ASD7 (*bph2*), Rathu Heenati (*Bph3, Bph17*), Babawee (*bph4*), Balamawee (*Bph27*+QTLs)	Swollen abdomen tests, survival
BPH [78]	Japan – Hadano-66, Chicugo-89, Koshi-99, Koshi-13	1966, 1989, 1999, 2013	Reiho	Monogenic backcrosses homozygous for T65 or PTB33 at five QTL positions	Swollen abdomen tests, survival

1: Numbers indicate years in which planthoppers were collected; 2: test differentials varied between years as indicated

Table S3. Details of selection experiments conducted with Asian brown planthopper (BPH),whitebacked planthopper (WBPH), green leafhopper (GLH) and green rice leafhopper (GRL) on resistant rice varieties

Species and Source Populations[1]	Exposed Varieties	Founder Population Size (Length of Experiment in Generations)	Rate of Virulence Adaptation (Generations)[2]	Replicates	Improvements that Approach Fitness Measures on Susceptible Controls
BPH - 'Biotype 1' at IRRI (Philippines)[79]	Mudgo (*Bph1*), ASD7 (*bph2*)	10 nymphs (3)[3]	3	0	Feeding, survival and development
BPH - Wild populations from Central Agricultural Experiment Station (Japan) [80]	Mudgo (*Bph1*), F8 262 (*Bph1*), ASD7 (*bph2*)	NR (9)	6-8	0	Preference, population growth
BPH - 'Biotype 1' at IRRI (Philippines)[81]	Mudgo (*Bph1*), ASD7 (*bph2*)	10 nymphs (13)[4]	7-8	0	Survival, development and reproduction
BPH - Subcultures of 'Biotype 1'; 'Biotype 2'; and 'Biotype 3' from IRRI (Philippines)[82]	Mudgo (*Bph1*), ASD7 (*bph2*)	25 nymphs[8] (11)	8-11	0	Feeding, survival and development
BPH - Burdekin River Region, Queensland (Australia)[82]	TN1 (none)	25 nymphs[8] (11)	8	0	Feeding, survival and development
BPH - Zhejiang Province, (China: 1981)[54]	Mudgo (*Bph1*)	100 nymphs (14)	11	0	Survival and development
BPH - Chiaya Agricultural Experimental Station (Taiwan)[83]	TN1 (none), Mudgo (*Bph1*), H 105 (*bph2*), Rathu Heenati (*Bph3*++)	50 nymphs (12)	6	0	Feeding, survival and development
BPH - Jatisari Field Laboratory, West Java (Indonesia)[25]	IR42 (*bph2*)	197 nymphs (5)	5	Individually reared (<40)	Survival and development
BPH - Laguna rice fields, Mindanao fields of IR36 (M36) and IR42 (M42)(Philippines)[84]	Laguna population on ASD7 (*bph2*), M36 on IR36 (*bph2*), M42 on IR42 (*bph2*)	200 gravid females (80 on ASD7, 22 on IR36 and IR42)	NR	0	Feeding, survival, weight gain, population growth, plant damage

BPH - Central Agriculture Experiment Station at Koonosu, Saitama (Japan: 1975)[85]	Norin-PL3 (*Bph1*); Norin-PL4 (*bph2*); Norin-PL7 (*bph4*)	400 nymphs (12)	5	0	Survival and development
BPH - Banaue; Nueva Ecija; IRRI (Philippines: 1996)[86]	IR22 (S); IR26 (*Bph1*); IR64 (*Bph1+*); IR72 (*Bph3*)	25 nymphs (15)	5-6 (IR22, IR26) 15+ (IR64, IR72)	3	Feeding, survival and development
BPH - Kyushu National Agricultural Extension Experiment Station and Hyogo Prefectural Agricultural Research Centre - mixed (Japan)[87,88]	Thai Col. 11 (*bph8*); Pokkali (*Bph9*)	200 nymphs (28)	10+	0	Survival, development, and reproduction
BPH - Dongjin (Korea: 1998) [89]	Chungchungbyeo (*Bph1*)	NR?? (6)	<3	0	Survival, development, and reproduction
BPH - Nanning and Hangzhou (China)[90]	TN1 (none), IR26 (*Bph1*), ASD7 (*bph2*)	NR (4)	4	NR	Survival, longevity, fecundity
BPH - Dangjin (Korea: 2005)[68]	Cheongcheongbyeo (*Bph1*); ASD7 (*bph2*)	NR [7 (Cheong); 3 (ASD7)]		0	Survival and development
BPH - TN1 colony at IRRI, admixed with field collected individuals (Philippines)[91]	Mudgo (*Bph1*); ASD7 (*bph2*)	500 adults (11)	Possibly already adapted	0	Nymph weight gain, nitrogen content, amino acid molar concentrations, proportional composition of amino acids
BPH – Not reported (China)[92]	Minghui 63 (S); Minghui 63 + Bph15 (*Bph15*)	<25 nymphs (7)	7+ (significant improvement after 1 generation)	5	Survival and development, no improvement in reproduction
BPH - North Luzon, Laguna, Bicol, Mindoro, Iloilo, Mindanao (Philippines: 2009)[93]	IR22 (none), IR65482-4-136-2-2 (*Bph10*); IR62 (*Bph3*); PTB33 (*bph2*, *Bph3+*)	100 adults (20)	10 (IR22, IR62), 15 (IR65482), 20+ (PTB33)	6	Feeding, adult weight and reproduction

WBPH – Not reported (China) [94]	ARC10239 (*wbph2*); N'Diang (*Wbph5*)	NR (15)	<15 (ARC10239), 15+ (N'Diang)	0	Feeding; Improved survival and development on ARC10239 but not on N'Diang, reproduction improved
GLH - Penang and Kelantan State (Malaysia: 1980)[95]	IR42 (*glh4*)	>1000 nymphs (4 or 5)[3]	4	4	Survival, development and reproduction
GLH - MARDI, Bumbong Lima (Malaysia)[96]	Pankhari 203 (*Glh1*); IR42 (*glh4*), IR28 (*Glh9*)	2000 (Pankhari 203); 1000 (IR42); 100 (IR28) nymphs (4)	2 (IR28) to 3 (Pankhari 203, IR42)	0	Population growth
GLH - 1500 pairs (male and female), 100 each from 15 sites in the (Philippines)[97]	Staggered design - Pankhari 203 (*Glh1*), IR8 (*Glh3*), Ptb8 (*glh4* - Susceptible), TAPL796 (*Glh6*), Moddai Karuppin (*Glh7*)	500 adult pairs (20)	9 (Pankhari203), 4-15 (IR8), 10 (TAPL796); 6-20 (Monddai Karuppin)[6]	0[7]	Survival, development and reproduction
GLH - 100 adults: South Cotabato (Philippines: 1985)[98]	IR26 (none), IR36 (*Glh10*), IR54 (*Glh9*), IR56 (*Glh9*), IR64 (none), TN1 (none)	50 adult females (15)	15+	0	Feeding, survival and development
GLH - Laguna (Philippines: 2008), Batangas, Quezon, Rizal, San Pablo (Philippines: 2010)[99,100]	Grh2-NIL (S); Grh4-NIL (S); Grh2+4-NIL (R); T65 (S)	400 to 1000 mixed adults and nymphs (10)	10 [99]; 26 [100]	5	Survival, development and population growth; but no improvement in reproduction
GRH - Hokuriku Research Centre (Japan: 1993)[101,102]	Nipponbare (S); Saikai 164 (R); Saikai 182 (*Grh2*); Kanto-PL 6 (?); [Norin-PL 5 (*Grh2+4*); Norin PL 6 (*Grh2+4*) not completed beyond third generation]	150-200 nymphs (8)	8	0	Survival, development and reproduction

1: Year of collection indicated where available; 2: Based on least adapted trait; 3: Returned to susceptible varieties for multiplication/oviposition; 4: Returned to susceptible for multiplication/oviposition during first three generations; 5: Only first 10 nymphs to emerge as adults were passed to successive generations; 6: staggered colonies were evaluated together during the 10th, 15th and 20th generations, but were not replicated for each observation

Table S4. Information on the nature of virulence adaptation in the brown planthopper (BPH),whitebacked planthopper (WBPH), green leafhopper (GLH) and green rice leafhopper (GRL) based on post-selection studies. See Figure 3 for further details.

**Herbivore Species - Gene**	**Natal Hosts**	**Maintains Virulence after returning to Susceptible/Other Variety**	**Gains Virulence against Other Rice Varieties with the Same Gene**	**Gains Virulence to Pyramided Lines Containing the Same Gene[1]**	**Gains Virulence to Lines with Different Resistance Genes**
BPH – *Bph1* [103]	F8 262 & F8 233 (NILs for *Bph1*)	-	Yes (Mudgo)	-	No (ASD7, *bph2*)
BPH – *Bph1* [103]	Mudgo	Yes (4 generations)	-	-	-
BPH – *Bph1* [80]	Mudgo, F8 262 (*Bph1*)	Yes (? Details)	Yes (? Details – reciprocal tests) preference, but not performance for *Bph1*		
BPH – *Bph1* [82]	Mudgo	Yes (maintains *Bph1* virulence for >10 generations)	-	-	Yes (ASD7, *bph2*)
BPH – *Bph1* [81]	Mudgo	-	-	-	No (ASD7, *bph2*)
BPH – *Bph1* [54]	Mudgo	-	-	-	Yes (ASD7, *bph2*)
BPH – *Bph1* [83]	Mudgo	-	Yes (IR26)	-	Yes (H105 & ASD7 [*bph2*])[2]
BPH – *Bph1* [104]	Mudgo				No (ASD7 [*bph2*])
BPH – *Bph1* [105]	Norin-PL3	-	-	-	No (Norin-PL7, *bph4*) preference tests
BPH – *Bph1* [89]	Chungchungbyeo	-	Yes (Mudgo, IR26, IR64)	No (Gayabyeo [*Bph1* + *bph2*])	No (Miryang 63 [*bph2*])
BPH – *Bph1* [106]	Mudgo				No (*BPH1/9-2*, *BPH1/9-7*, *BPH1/0-9*)[4]
BPH – *bph2* [80]	ASD7	Yes (? Details)			
BPH – *bph2* [81]	ASD7	-	-	-	No (Mudgo, *Bph3*)
BPH – *bph2* (A) [83]	H105	-	Yes (ASD7)	-	No (Babawee & Thirissae , *bph4*)[2]
BPH – *bph2* (B)	H105				No (Bph1)
BPH – *bph2* (C)	H105				No (Rathu Heenati, *Bph3*)
BPH – *bph2* [104]	ASD7				No (Mudgo [*Bph1*])

BPH – *bph2* (A) [84]	ASD7, IR36, IR42		Yes (reciprocal tests) – not uniform - depends on natal host and exposed variety		No (IR46 [*Bph1*],
BPH – *bph2* (B)	ASD7, IR36, IR42				No (IR62 [*Bph3*])
BPH – *bph2* [105]	Norin-PL4	-	-		Yes (Norin-PL3, *Bph1*) preference tests
BPH – *bph2* [107]	ASD7	No (lost virulence after 7 generations on TN1)[2]			
BPH – *bph2* [106]	ASD7				No (*BPH1/9-1*, *BPH1/9-7*, *BPH1/9-9*)[4]
BPH – *Bph3*++ [83]	Rathu Heenati	-	Yes (Muthunanikan - *Bph3*)	-	Slight (Babawee & Thirissae [*bph4*])*
BPH – *Bph3* [108]	IR62	-	Yes (IR60, IR70, IR72, Rathu Heenati - depends on varieties with the gene)	Yes (PTB33)	Yes (IR66 & Babawee [*bph4*])
BPH – *Bph3* [109]	IR62	-	-	Yes (PTB33)	Yes (IR65482-4, [*Bph10*])
BPH – *Bph3* (A) [110]	IR62	-	Yes (Rathu Heenati)	Yes (PTB33/Rathu Heennati)	Yes (Babawee [*bph4*])[3]
BPH – *Bph3* (B)	IR62				Yes (Balamawee [*Bph9*])[3]
BPH – *Bph3* (C)	IR62				Yes (IR65482-7 [*Bph18*])[3],
BPH – *Bph3*++ [110]	PTB33	-	Yes (Rathu Heenati)	-	Yes (Balamawee [*Bph9*])
BPH – *bph4* (A) [105]	Norin-PL7	-	-	-	No (Norin-PL3, *Bph1*) but not well adapted to natal host
BPH – *bph4* (B)	Norin-PL7				No (Norin-PL4, *bph2*) but not well adapted to natal host
BPH – *bph8* [87]	Thai Col. 11	-	Yes (Thai Col. 5)	-	Slight (Pokkali, Balamawee [*Bph9*])
BPH – *Bph9* [87]	Pokkali	-	Slight (Balamawee)	-	Yes (Thai Col. 11, Thai Col. 5 [*bph8*])
BPH – *BPH10* (A) [109,110]	IR65482-4	-	-	-	Yes IR62, [*Bph3*]
BPH – *BPH10* (B)	IR65482-4	-	-	-	Yes (PTB33 [*Bph3*, *bph2*++])
WBPH – *wbph2* [94]	ARC10239	-	-	-	No (Rathu Heenati [*Bph3*++])

GLH – *Glh1* [111]	Pankhari203		Yes (varies depending on minor genes)		
GLH – *Glh3* [111]	IR8		Yes (varies depending on minor genes)		
GLH – *Glh6* [112]	TAPL #796	-	-	-	No (IR36, [*Glh10*])
GLH – *Glh10* (A) [98]	IR36	-	-	-	Yes (IR54 [*Glh9*]);
GLH – *Glh10* (B)	IR36				No (IR62 [*Bph3*])
GLH – *Grl2* [100]	*GRL2*-NIL			Yes (*GRL2+4*-NIL), performance, but not preference	
GLH – *Grl4* [100]	*GRL4*-NIL			Yes (*GRL2+4*-NIL), preference and performance	
GLH – *Grl2* + *Grl4* [100]	*GRL2+4*-PYL	Yes (6 generations), increased survival and weight gain of adults			
GRL – *Grl2* (A) [101]	Saikai 182	-	Yes (Saikai164)	No (Norin-PL5, Norin-PL6 [*Grh2* + *Grh4*])	No (IR24, Norin-PL2, Chugoku 105 [*Grh1*],
GRL – *Grl2* (B)	Saikai 182				No Aichi 80 [*Grl3*t])
GRL – *Grl3*(t) [101]	Aichi 80, Kanto-PL6	-	Yes (Aichi 80, Kanto-PL6, Tadukan, Rantajandemas2)	-	No (Saikai 164, Saikai 182 [*Grh2*]

1: includes partial improvements in fitness; 2: reared on ASD7 for only 4 generations; 3: effects not apparent in 60-day old plants; 4: tested against different alleles of the *BPH1/9* gene

Table S5. Advances in understanding the mechanisms of virulence adaptation in the brown planthopper (BPH) and green leafhopper (GLH)

**Herbivore - Category**	**Year**	**Exposed host (genes)**	**Populations**	**Test populations**	**Replicates**	**Fitness test/trait**	**Mechanism**
BPH - Molecular or Biochemical [113]	2017	YHY15 (*Bph15*)	Full-sib mated biotype 1 and biotype Y	Planthoppers feeding on artificial diet with ethanol extracts of host varieties	NR	Adult weight gain, honeydew, EPG	P450s play a role in feeding by adapted BPH on diets with extracts from resistant rice
BPH - Epigenetic [114]	2022	Rathu Heenati (*Bph3*)	Colony reared on TN1 for 30 generations	Reared on Rathu Heenati for 4 generations	?	Not reported	Epigenetic modifications including DNA methylation, which is heritable, But unstable after return to susceptible host
BPH - Genetic [115]	1979	Mudgo (*Bph1*), H105 (*bph2*)	Biotypes 1, 2 and 3	Reciprocal crosses and Backcrosses	?	Infesting and wilting of plants	Ability to infest Mudgo controlled by a recessive gene, ability to infest H105 controlled by a dominant gene
BPH - Genetic [116]	1981	Mudgo (*Bph1*), ASD7 (*bph2*)	Biotypes 1, 2 and 3	Reciprocal crosses and Backcrosses	0	Survival to adult	Adult emergence of biotype 2 (Mudgo) controlled by one incomplete dominant gene; adult emergence in biotype 3 (ASD7) controlled by one incomplete recessive gene
BPH - Genetic [21]	1984	IR42 (*bph2*)	North Sumatra (biotype3), Bogor (biotype 1)	Reciprocal crosses and Backcrosses	0 (20 individuals)	Nymphal development; female longevity	Virulence associated with recessive genes; some indication of female-linked virulence

BPH - Genetic [83]	1985	Mudgo (*Bph1*), H105 (*bph2*)	Biotypes I, II and III	Reciprocal crosses and Backcrosses	0 (10-40 individuals)	Nymph survival	Survival of biotype 2, recessive gene; survival of biotype 3, partly dominant, no indication of sex-linked responses; assumed polygenic inheritance and dominance of genetically controlled traits with some biotypes; therefore, complex genetics of biotypes; virulence to Mudgo and H105 not controlled by major genes; virulence controlled by many genes
BPH - Genetic [117]	1999	Saikai 190 (*Bph1*), ASD7 (*bph2*)	Field population (Kumamoto, Japan)	Full-sib families	0 (based on tests with individuals)	Honeydew excretion, oviposition	Biotype 3 - binomial distribution of honeydew production/excretion; therefore a threshold character with virulent and avirulent individuals; similar contributions by both parents; low genetic correlation between virulence to Saikai 190 and ASD7 - i.e., distinct genetic mechanisms
BPH - Genetic [118]	2014	Mudgo (*Bph1*)	Biotypes 1 and 2	Full-sib inbred	Tests with individuals	Host preference, nymph survival, development and weight gain	High density linkage map of BPH, to identify loci involved in virulence against *Bph1*; *Qhp7* locus associated with preference, *Qgr5* and *Qrg14* loci associated with nymph growth

BPH - Genetic [119]	2014	Saikai190 (*Bph1*)	Izumo87 on Koshihikari ; Chikuo89 on Saikai190 - full-sib crossings for >10 generations	Full-sib inbred	Tests with individuals	Honeydew excretion	Bulk segregant analyses of F2 progeny revealed virulence associated with a single recessive gene (*vBph1*)
BPH - Genetic [120]	2024	IR36 (*bph2*)	Imidacloprid-resistant population at Guangdong Academy of Agricultural Sciences and field samples from three sites in Guangdong province	Monitoring of colonies and field populations	0 for selected colonies, 3+ for field samples	Oviposition	Mutations in nAChR-7-like and CYP4C61 insecticide resistance genes accelerated adaptation to IR36 (*bph2*) and correlations between these mutations and virulence in field populations
BPH - Microbiome [121]	2010	Mudgo (*Bph1*), ASD7 (*bph2*)		Biotypes 1, 2 and 3	0	Not reported	Bacteria-like organisms associated with saliva - orally secreted microbes; BPH on different hosts have different microbiomes
BPH - Microbiome [122]	2015	Mudgo (*Bph1*), ASD7, IR42 (*bph2*), IR56 (*Bph3*)	Colonies reared on TN1, Mudgo, ASD7, IR42 and IR56; four field collected populations (from China, Vietnam and the Philippines) reared on TN1	Monitoring of colonies on TN1, ASD7 and IR56 over 30 generations	0, 4 for TN1	Not reported	Some association of bacteria with selection hosts - *Arsenophonus* more abundant on TN1, infection frequency increased on TN1 and IR56 over selection, but not on IR42
BPH - Microbiome [123]	2015	Mudgo (*Bph1*), ASD7 (*bph2*)	Biotypes 1, 2 and 3	Assessment of 5 bacteria on 3 varieties after 140 generations, assessment of changes during selection of biotype1 on Mudgo and ASD7	0	Not reported	*Arsenophonus* and *Serrantia* possibly related to virulence against *Bph1* and *bph2*; increase in *Chryseobacterium* between generations 1 and 2 on ASD7, and between 1 and 3 on Mudgo, no consistent patterns for other bacteria

BPH - Microbiome [124]	2019	IR36 (*bph2*), Rathu Heenati (*Bph3*++)	Field population (Guangzhou, China)	Monitoring of colonies on TN1, IR36 and Rathu Heenati over 16 generations	0	Not reported	Microbiomes differed between planthoppers from the colonies; *Asaia* sp. suggested important in adaptation, found in eggs and in egg-infested rice
BPH - Microbiome [125]	2021	ASD7 (*bph2*)	Adapted colonies on TN1 and ASD7 for 50 generations	Newly emerged adults (male and female)	0	Not reported	Microbiomes are different - species richness and gut microbial profiles
BPH - Microbiome [126]	2021	NR	Biotype 1	Antibiotic treated and untreated	0	Not reported	Microbiome perturbation affects rice defense/induced responses during feeding by BPH
BPH - Microbiome [127]	2024	NR	Field population (Guangzhou, China)	Females on diets with different secondary metabolites exposed over 5 days	NR	Female longevity, weight gain and fecundity	BPH feed on artificial diet with methyl jasmonate had fewer *Ascomycete* endosymbionts - numbers decreased over time from 1-5 days
GLH - Microbiome [128]	2019	Grl2-NIL (*Grl2*), Grl4-NIL (*Grl4*), Grl2+4-PYL (*Grl2* + *Grl4*)	Colonies adapted to NILs and PYLs for 20 generations	Assessed at generation 20 and 26, rearing of Grl4 and Grl2+4 on Grl2+4-PYL for 6 generations	5	Nymph survival, biomass, adult longevity, biomass, eggs laid	Six bacteria (*Candidatus sulcus* clade, *Dyella*, *Bosea*, *Mycobacterium*, *Sandaracinus*, *Dyadobacter*) were associated with virulence (based on abundance or proportional occurrence) these also generally increased in abundance over 6 generations of selection on resistant hosts

BPH - YLS [90]	2004	Mudgo (*Bph1*), ASD7 (*bph2*)	Biotypes 1, 2 and 3	Monitoring of YLS in biotype 1 over 4 generations on TN1, Mudgo and ASD7	0	Survival, nymphal duration, fecundity	YLS abundance declined when nymphs were reared on resistant varieties; gradual increase in YLS abundance over generations as planthoppers adapted; YLS abundance was related to activity of alanine trasaminase and aspartic transaminase
BPH - YLS [91]	2011	Mudgo (*Bph1*), ASD7 (*bph2*)	Colonies (IRRI laboratory with introgression of field-caught individuals)	Monitoring over 11 generations of exposure to Mudgo and ASD7	0	Weight gain on symbiotic and aposymbiotic planthoppers	YSL and planthopper abilities to acquire rare amino acids improved over generations of selection - this was independent of YLS abundance
BPH - YLS [93]	2015	IR62 (*Bph3*), PTB33 (*Bph3*++), IR65482-4 (*BPH10*)	Field populations (6 sites in the Philippines)	Monitoring of YLS over 20 generations on differentials	6	Honeydew composition, eggs laid, adult weight	YLS densities not related to virulence or host resistance strength
BPH - YLS [109]	2017	IR62 (*Bph3*), PTB33 (*Bph3*++), IR65482-4 (*BPH10*)	Colonies selected on TN1, IR22, IR62, PTB33 and IR65482-4	Reciprocal feeding after selection and outbreeding	2	Nymph weight in symbiotic and aposymbiotic planthoppers	Depletion of YLS did not alter relative fitness of adapted populations across a range of hosts; little role for YLS densities in virulence adaptation and host plant switching
BPH - YLS [129]	2021	IR62 (*Bph3*)	Colonies selected on TN1 and IR62	Hybridization crosses and compatible and incompatible exposure	2	Honeydew composition, eggs laid, adult weight, nymph survival	Females made a greater contribution to virulence of progeny; only weak effects of YLS densities

BPH - YLS [130]	2022	Mudgo (*Bph1*), ASD7 (*bph2*), Rathu Heenati (*Bph3*++)	Field population (Guangzhou, China)	Monitoring of 3 symbiont types on each host for 8 generations	0	Not reported	*Ascomycetes*, *Pichia*-like and *Candida*-like endosymbionts decline in generations 1 and 2 on novel hosts, then increase - but proportions vary between host varieties

References

1. Horgan, F.G. Mechanisms of resistance: a major gap in understanding planthopper-rice interactions. In *Planthoppers: new threats to the sustainability of intensive rice production systems in Asia*, Heong, K.L., Hardy, B., Eds.; International Rice Research Institute: Los Baños, Philippines, 2009; pp. 281-302.
2. Horgan, F.G.; Mundaca, E.A.; Quintana, R.; Naredo, A.I.; Almazan, M.L.P.; Bernal, C.C. Efficacy and cost-effectiveness of phenotyping for rice resistance and tolerance to planthoppers. *Insects* **2021**, *12*, 847, doi:10.3390/insects12100847.
3. Kabir, M.A.; Alam, M.S. Varietal screening for resistance to brown planthopper and its biotype in Bangladesh. *IRRN* **1981**, *6-5*, 8-9.
4. Kalode, M.B.; Krishna, T.S. Varietal resistance to brown planthopper in India. In *Brown Planthopper: Threat to Rice Production in Asia*, International Rice Research Institute, Ed.; International Rice Research Institute: Los Baños, Philippines, 1979; pp. 187-200.
5. Pathak, P.K.; Lal, M.N.; Pant, G.B. Varietal resistance to the brown planthopper *Nilaparvata lugens* (Stål) and its biotypes. *IRRN* **1976**, *1-2*, 8.
6. Verma, S.K.; Pathak, P.K.; Singh, B.N.; Lal, M.N. Indian biotypes of the brown planthopper. *IRRN* **1979**, *4-6*, 7.
7. Baskaran, B.; Narayanaswamy, P.; Sambasivam, A. BPH outbreak in South Arcot District, Tamil Nadu, India. . *IRRN* **1983**, *8-4*, 18.
8. Veronica, B.K.; Kalode, M.B. Virulence in brown planthopper populations from different sources in Andhra-Prasesh. *Indian Journal of Agricultural Sciences* **1983**, *53*, 1084-1086.
9. Das, S.R.; Dhal, N.K.; Mohanty, H.K. IR13429-196-1-20 and IR17525-56-2-2-2: two promising brown planthopper (BPH)-tolerant lines. *IRRN* **1984**, *9-3*, 7.
10. Velusamy, R.; Chelliah, S.; Heinrichs, E.A.; Medrano, F. Brown planthopper biotypes in India. *IRRN* **1984**, *9-2*, 19.
11. Prakasa Rao, P.S. Testing for field resistance in rice under induced brown planthopper (BPH) outbreaks. *IRRN* **1985**, *10-4*, 5-6.
12. Velusamy, R.; Saxena, R.C. Genes conditioning resistance to brown planthopper (BPH). *IRRN* **1989**, *14-1*, 12-13.
13. Pophaly, D.J.; Rana, D.K. Virulence of brown planthopper (BPH) in Raipur, India. *IRRN* **1992**, *17-1*, 12-13.
14. Pophaly, D.J.; Rana, D.K. Reaction of IR varieties to the brown planthopper (BPH) population in Raipur, Madhya Pradesh, India. *IRRN* **1993**, *18-1*, 27-28.
15. Horgan, F.G.; Ramal, A.F.; Bentur, J.S.; Kumar, R.; Bhanu, K.V.; Sarao, P.S.; Iswanto, E.H.; Chien, H.V.; Phyu, M.H.; Bernal, C.C.; et al. Virulence of brown planthopper (*Nilaparvata lugens*) populations from South and South East Asia against resistant rice varieties. *Crop Protection* **2015**, *78*, 222-231, doi:10.1016/j.cropro.2015.09.014.
16. Priyadarshini, S.; Lakshmi, V.J.; Madhav, M.S.; Rajeswari, B.; Srinivas, C. Virulence of rice brown planthopper, *Nilaparvata lugens* (Stål) population from Khammam district of Telangana State against rice genotypes and its morphometrics. *Journal of Research PJTSAU* **2021**, *49*, 16-22.
17. Shrestha, G.L.; Adhikary, R.R. A new brown planthopper (BPH) biotype in Parwanipur, Nepal. *IRRN* **1987**, *12-3*, 34.
18. Fernando, H.; Senadhera, D.; Elikawela, Y.; de Alwis, H.M.; Kudagamage, C. Varietal resistance to the brown planthopper in Sri Lanka. In *Brown Planthopper: Threat to Rice Production in Asia*, International Rice Research Institute, Ed.; International Rice Research Institute: Los Baños, Philippines, 1979; pp. 241-250.
19. Mochida, O. IR26 found susceptible to the brown planthopper in North Sumatra, Indonesia. *IRRN* **1977**, *2-5*, 10-11.
20. Oka, I.N. Quick method for identifying brown planthopper biotypes in the field. *IRRN* **1978**, *3-6*, 11-12.
21. Sogawa, K.; Kilin, D. Inheritance of virulence of the North Sumatra population of the brown planthopper (BPH) on IR42. *IRRN* **1984**, *9-6*, 14-15.
22. Sogawa, K.; Kusumayadi, A. Monitoring brown planthopper (BPH) biotypes by rice garden in North Sumatra. *IRRN* **1984**, *9-6*, 15-16.
23. Sogawa, K.; Kilin, D.; Bhagiawati, A.H. Characterization of the brown planthopper population on IR42 in North Sumatra, Indonesia. *IRRN* **1984**, *9-1*, 25.
24. Sogawa, K.; Soekirno; Raksadinata, Y. New genetic makeup of brown planthopper (BPH) populations in Central Java, Indonesia. *IRRN* **1987**, *12-6*, 29-30.
25. Sogawa, K.; Kilin, D. Biotype shift in a brown planthopper (BPH) population on IR42 *IRRN* **1987**, *12-4*, 40.

26. Erdiansyah, I.; Damanhuri. Performance of resistance of rice varieties recommendation of Jember Regency to brown planthopper pest (*Nilaparvata lugens* Stål.). *IOP Conference Series: Earth and Environmental Science* **2018**, *207*, 012041.
27. Chaerani; Damayanti, D.; Trisnaningsih; Yuriyah, S.; Kusumanegara, K.; Dadang, A.; Sutrisno, S.; Bahagiawati. Virulence of brown planthopper and development of core collection of the pest. *Jurnal Penelitian Pertanian Tanaman Pangan* **2016**, *35*, 125601.
28. Habibuddin, H. Variation of brown planthopper population from major rice regions of Peninsular Malaysia. *MARDI Research Journal* **1989**, *17*, 218-224.
29. Ito, K.; Wada, T.; Takahashi, A.; Salleh, N.; Hassim, H. Brown planthopper *Nilaparvata lugens* Stål (Homoptera, Delphacidae) biotypes capable of attacking resistant rice varieties in Malaysia. *Applied Entomology and Zoology* **1994**, *29*, 523-532, doi:10.1303/aez.29.523.
30. Wada, T.; Ito, K.; Takahashi, A. Biotype comparisons of the brown planthopper, *Nilaparvata lugens* (Homoptera: Delphacidae) collected in Japan and the Indochina Peninsula. *Applied Entomology and Zoology* **1994**, *29*, 477-484, doi:10.1303/aez.29.477.
31. Feuer, R. Biotype 2 brown planthopper in the Philippines. *IRRN* **1976**, *1-1*, 15.
32. Domingo, I.T. Pest incidence in Central Luzon, the rice granary of the Philippines. *IRRN* **1976**, *1-2*, 9.
33. Heinrichs, E.A.; Viajante, V. Field reaction of rice to the brown planthopper and ragged stunt virus. *IRRN* **1978**, *3-4*, 9-10.
34. Peralta, C.A.; Fontanilla, W.S.; Ferrer, L.S. Brown planthopper resurgence on IR36 in Mindanao, Philippines. *IRRN* **1983**, *8-2*, 13-14.
35. Medrano, F.; Heinrichs, E.A. Responses of resistant rices to brown planthoppers (BPH) collected in Mindanao, Philippines. . *IRRN* **1985**, *10-6*, 14-15.
36. Joshi, R.C.; Shepard, B.M.; Kenmore, P.E.; Lydia, R. Insecticide-induced resurgence of brown planthopper (BPH) on IR62. *IRRN* **1992**, *17-3*, 9-10.
37. Medina, E.B.; Bernal, C.C.; Cohen, M.B. Role of host plant resistance in successful control of brown planthopper in Central Luzon, Philippines. *IRRN* **1996**, *21-2*, 53.
38. Chansrisommai, N.; Katanyukul, W. Resistance of modern rice varieties to the brown planthopper in Thailand. *IRRN* **1982**, *7-4*, 9.
39. Thanysiriwat, T.; Pattwatang, P.; Angeles, E.R. New biotypes of brown planthopper in Thailand. In Proceedings of the Rice and Temperate Cereal Crops Annual Conference, Chon Buri, Thailand, 9-11 June 2009, Bangkok, Thailand, 2009; pp. 386-389.
40. Chaiyawat, P. Virulence of brown planthopper (*Nilaparvata lugens* Stål) against differential resistant and certified rice varieties in the major irrigated rice-growing areas of Thailand. In Proceedings of the 28th International Rice Research Conference, 8-12 November 2010, Hanoi, Vietnam, 2010; pp. 1-9.
41. Sreewongchai, T.; Worede, F.; Phumichai, C.; Sripichitt, P. Evaluation of rice genotypes for resistance to brown planthopper (*Nilaparvata lugens* Stål) populations from the central region of Thailand. *Agriculture and Natural Resources* **2015**, *49*, 506-515.
42. Huynh, N.V. New biotype of brown planthopper in the Mekong Delta of Vietnam. *IRRN* **1977**, *2-6*, 10.
43. Thuat, N.C.; Thans, D.V. Population dynamics of the brown planthopper (BPH) in the Mekong Delta. *IRRN* **1984**, *9-5*, 14-15.
44. Huynh, N.V.; Nhung, H.T. High virulence of new brown planthopper (BPH) populations in the Mekong Delta, Vietnam. *IRRN* **1987**, *13-5*, 16.
45. Chau, L.M. Virulence of a new biotype of brown planthopper (BPH) in Mekong Delta. *IRRN* **1990**, *17-1*, 14-15.
46. Chau, L.M. Development of a brown planthopper (BPH) biotype and change in varietal resistance in Mekong Delta. *IRRN* **1990**, *15-5*, 12.
47. Thuat, N.C.; Huong, N.T.; Binh, D.T.; Chien, H.V.; Chau, N.L. Virulence of brown planthopper (BPH) in Vietnam. *IRRN* **1992**, *17-2*, 11.
48. Chau, N.L.; Thuat, N.C.; Chai, V.T. Changes in brown planthopper (BPH) biotypes in the Mekong Delta of Vietnam. *IRRN* **1993**, *18*, 26-27.
49. Phuong, L.T.; Chau, L.M. Resistance of varieties derived from *Oryza sativa/Oryza officinalis* to brown planthopper in the Mekong Delta, Vietnam. *IRRN* **1997**, *22-1*, 26-27.

50. Stapley, J.; May-Jackson, Y.Y.; Golden, W. Varietal resistance to the brown planthopper in the Solomon Islands. In *Brown Planthopper: Threat to Rice Production in Asia*, International Rice Research Institute, Ed.; International Rice Research Institute: Los Banõs, Philippines, 1979; pp. 233-239.
51. Ho, D.T. Effect of sequential release of resistant rices on brown planthopper (BPH) biotype development in the Solomon Islands. *IRRN* **1985**, *10-4*, 16-17.
52. Ho, D.T.; Taro, A. Rice resistance to brown planthopper (BPH) in the Solomon Islands. *IRRN* **1985**, *10-2*, 6-7.
53. Wu, J.-T.; Qiu, X. Screening rice for brown planthopper (BPH) resistance. *IRRN* **1984**, *9-3*, 6.
54. Wu, G.R.; Chen, F.Y.; Tao, L.Y.; Huang, C.W.; Fen, B.C. Studies on the biotypes of the brown planthopper, *Nilaparvata lugens* (Stål). *Acta Entomologica Sinica* **1983**, *26*, 154-160.
55. Lei, H.; Liu, G.; Wu, M.; Tian, J. Varietal screening for brown planthopper resistance in China. *IRRN* **1984**, *9-1*, 1-12.
56. Lei, H.; Liu, G.; Wu, M.; Jiang, J. Biotype populations of *Nilaparvata lugens* in Hunan, China. *IRRN* **1987**, *12-5*, 22-23.
57. Yu, X.; Lu, Z.; Wu, G.; Tao, L.; Chen, J.; Zheng, X.; Xu, H. The biotypes, wing-forms and the immigration of brown planthopper, *Nilaparvata lugens* Stål, in Zhejiang Province, China. *Journal of Asia-Pacific Entomology* **2001**, *4*, 201-207, doi:https://doi.org/10.1016/S1226-8615(08)60124-7.
58. Lu, Z.; Yu, X.; Wu, G.; Tao, L.; Chen, J.; Zheng, X. The virulence change and damage characteristics of various geographic populations of brown planthopper. *Insect Science* **1999**, *6*, 146-154, doi:https://doi.org/10.1111/j.1744-7917.1999.tb00161.x.
59. Zhang, Y.; Tan, Y.; Huang, B. Monitoring variation in brown planthopper biotype in Guangdong, China. *IRRN* **1995**, *20-4*, 19-20.
60. Yu, X.; Wu, G.; Tao, L. Virulence of brown planthopper (BPH) populations collected in China. *IRRN* **1991**, *16-3*, 26.
61. Takahashi, A.; Ito, K.; Tang, J.; Hu, G.W.; Wada, T. Biotypical property in the populations of the brown planthopper, *Nilaparvata lugens* Stål (Homoptera, Delphacidae), collected in China and Japan. *Applied Entomology and Zoology* **1994**, *29*, 461-463, doi:10.1303/aez.29.461.
62. Li, B.; He, J.; Wan, P.; Lai, F.; Wang, W.; Sun, Y.; Jiang, F.Q.; Chen, B.; Fu, Q. Studies on the virulence of different geographic populations of *Nilaparvata lugens* (Stål) collected from China. *Journal of Environmental Entomology* **2019**, *41*, 9-16.
63. Chen, L.C.; Chang, W.L. Inheritance of resistance to brown planthopper in rice variety, Mudgo. *Journal of Taiwan Agr. Res.* **1971**, *20*, 57-60.
64. Chang, W.L. Tainung 68, the first BPH-resistant *japonica* cultivar developed in Taiwan. *IRRN* **1982**, *7-4*, 8.
65. Sogawa, K. Rice brown planthopper (BPH) immigrants in Japan change biotype. *IRRN* **1992**, *17-2*, 26-27.
66. Tanaka, K.; Matsumura, M. Development of virulence to resistant rice varieties in the brown planthopper, *Nilaparvata lugens* (Homoptera : Delphacidae), immigrating into Japan. *Applied Entomology and Zoology* **2000**, *35*, 529-533, doi:10.1303/aez.2000.529.
67. Lippold, P.C.; Lee, J.O.; Kim, Y.H.; Park, J.; Chung, K.H.; Davis, M.D.; Steenberg, K. Feeding of brown planthopper on rice varieties labled with 32P. *IRRN* **1978**, *3-4*, 8-9.
68. Seo, B.Y.; Jung, J.K.; Choi, B.R.; Park, H.M.; Lee, S.W.; Lee, B.H. Survival rate and stylet penetration behavior of current Korean populations of the brown planthopper, *Nilaparvata lugens*, on resistant rice varieties. *Journal of Asia-Pacific Entomology* **2010**, *13*, 1-7, doi:10.1016/j.aspen.2009.09.001.
69. Lee, J.O.; Goh, H.G.; Kim, C.C.; Park, J.S. Brown planthopper biotypes in Korea. *IRRN* **1983**, *8-5*, 15.
70. Lee, J.O.; Goh, H.G.; Kim, Y.H.; Kim, C.G. Brown planthopper-resistant *japonica* varieties developed in Korea. *IRRN* **1983**, *8-6*, 5.
71. Lee, J.O.; Goh, H.G. Yield losses of a susceptible rice variety to brown planthopper (BPH) in Korea. *IRRN* **1984**, *9-4*, 9.
72. Sogawa, K. Shifts in population characteristics of brown planthopper (BPH) immigrants to Japan. *IRRN* **1993**, *18-3*, 35-36.
73. Tanaka, K. A simple method for evaluating the virulence of the brown planthopper. *IRRN* **2000**, *25-1*, 18-19.
74. Tanaka, K. Recent status in virulence to resistant rice varieties of brown planthopper *Nilaparvata lugens* immigrating into Japan. *Annual Report of the Kanto-Tosan Plant Protection Society* **1999**, *46*, 85-88.
75. Myint, K.K.M.; Matsumura, M.; Takagi, M.; Yasui, H. Demographic parameters of long-term laboratory strains of the brown planthopper, *Nilaparvata lugens* Stål, (Homoptera: Delphacidae) on resistance genes, *bph20*(t) and *Bph21*(t) in rice. *Journal of the Faculty of Agriculture Kyushu University* **2009**, *54*, 159-164.

76. Myint, K.K.M.; Yasui, H.; Takagi, M.; Matsumura, M. Virulence of long-term laboratory populations of the brown planthopper, *Nilaparvata lugens* (Stål), and whitebacked Planthopper, *Sogatella furcifera* (Horvath) (Homoptera: Delphacidae), on rice differential varieties. *Applied Entomology and Zoology* **2009**, *44*, 149-153, doi:10.1303/aez.2009.149.
77. Fujii, T.; Yoshida, K.; Kobayashi, T.; Myint, K.K.M.; Yasui, H.; Sanada-Morimura, S.; Matsumura, M. Long-term virulence monitoring of differential cultivars in Japan's immigrant populations of *Nilaparvata lugens* (Hemiptera: Delphacidae) in 2001-2019. *Applied Entomology and Zoology* **2021**, *56*, 407-418, doi:10.1007/s13355-021-00749-3.
78. Tabata, S.; Yamagata, Y.; Fujita, D.; Sanada-Morimura, S.; Matsumura, M.; Yasui, H. Genetic Dissection of the Breakdown of Durable Resistance in *indica* Rice Variety PTB33 to Brown Planthoppers *Nilaparvata Lugens* (Stål). Research Square (Preprint). Available online: https://doi.org/10.21203/rs.3.rs-1131675/v1 (accessed on 1 August 2024).
79. Den Hollander, J.; Pathak, P.K. The genetics of the 'biotypes' of the rice brown planthopper, *Nilaparvata lugens*. *Entomologia Experimentalis et Applicata* **1981**, *29*, 76-86, doi:https://doi.org/10.1111/j.1570-7458.1981.tb03044.x.
80. Ito, K.; Kisimoto, R. Selection of new biotypes of the brown planthopper, *Nilaparvata lugens* Stål, capable of surviving on resistant rice cultivars. *Journal of the Central Agricultural Experiment Station* **1981**, *1*, 139-154.
81. Pathak, P.K.; Heinrichs, E.A. Selection of biotype populations 2 and 3 of *Nilaparvata lugens* by exposure to resistant rice varieties. *Environmental Entomology* **1982**, *11*, 85-90, doi:10.1093/ee/11.1.85.
82. Claridge, M.F.; Den Hollander, J. Virulence to rice cultivars and selection for virulence in populations of the brown planthopper *Nilaparvata lugens*. *Entomologia Experimentalis et Applicata* **1982**, *32*, 213-221, doi:10.1111/j.1570-7458.1982.tb03208.x.
83. Cheng, C.-H. Interactions between biotypes of the brown planthopper and rice varieties. *Journal of Agricultural Research China* **1985**, *34*, 299-314.
84. Bahagiawati, A.; Heinrichs, E.; Medrano, F. Effect of host plant on the level of virulence of *Nilaparvata lugens* (Homoptera: Delphacidae) on rice cultivars. *Environmental Entomology* **1989**, *18*, 489-493.
85. Nemoto, H.; Yokoo, M. Experimental selection of a brown planthopper population on mixtures of resistant rice lines. *Japanese Journal of Breeding* **1994**, *44*, 133-136, doi:10.1270/jsbbs1951.44.133.
86. Alam, S.N.; Cohen, M.B. Durability of brown planthopper, *Nilaparvata lugens*, resistance in rice variety IR64 in greenhouse selection studies. *Entomologia Experimentalis et Applicata* **1998**, *89*, 71-78, doi:10.1046/j.1570-7458.1998.00383.x.
87. Ketipearachchi, Y.; Kaneda, C.; Nakamura, C. Adaptation of the brown planthopper (BPH), *Nilaparvata lugens* (Stål) (Homoptera : Delphacidae), to BPH resistant rice cultivars carrying *bph8* or *Bph9*. *Applied Entomology and Zoology* **1998**, *33*, 497-505, doi:10.1303/aez.33.497.
88. Ketipearachchi, Y.; Nakamura, C.; Kaneda, C. Biotype shifting of brown planthopper (BPH) on rice cultivars. *Kinki Journal of Crop Science and Breeding* **1997**, *42*, 7-9.
89. Hwang, I.; Kim, J.; Song, Y. Changes in the fitness of brown planthopper, *Nilaparvata lugens* Stål (Homoptera: Delphacidae) to several resistant rice varieties after multi-generational selection. *Korean Journal of Applied Entomology* **2002**, *41*, p113-121.
90. Lu, Z.-X.; Yu, X.-P.; Chen, J.-M.; Zheng, X.-S.; Xu, H.-X.; Zhang, J.-F.; Chen, L.-Z. Dynamics of yeast-like symbiote and its relationship with the virulence of brown planthopper, *Nilaparvata lugens* Stål, to resistant rice varieties. *Journal of Asia-Pacific Entomology* **2004**, *7*, 317-323, doi:https://doi.org/10.1016/S1226-8615(08)60233-2.
91. Chen, Y.H.; Bernal, C.C.; Tan, J.; Horgan, F.G.; Fitzgerald, M.A. Planthopper "adaptation" to resistant rice varieties: Changes in amino acid composition over time. *Journal of Insect Physiology* **2011**, *57*, 1375-1384, doi:10.1016/j.jinsphys.2011.07.002.
92. Li, J.; Shang, K.; Liu, J.; Jiang, T.; Hu, D.; Hua, H. Multi-generational effects of rice harboring *Bph15* on brown planthopper, *Nilaparvata lugens*. *Pest Management Science* **2014**, *70*, 310-317, doi:https://doi.org/10.1002/ps.3560.
93. Ferrater, J.B.; Naredo, A.I.; Almazan, M.L.P.; de Jong, P.W.; Dicke, M.; Horgan, F.G. Varied responses by yeast-like symbionts during virulence adaptation in a monophagous phloem-feeding insect. *Arthropod-Plant Interactions* **2015**, *9*, 215-224, doi:10.1007/s11829-015-9373-0.
94. Shen, J.-h.; Wang, Y.; Sogawa, K.; Hattori, M.; Liu, G.-j. Monitoring the changes in virulence of different populations of the whitebacked planthopper, *Sogatella furcifera* rearing on resistant rice varieties. *Chinese Journal of Rice Science* **2003**, *17*, 84.
95. Kobayashi, A.; Supaad, M.A.; Othman, B.O. Inheritance of resistance of rice to tungro and biotype selection of green leafhopper in Malaysia. *JARQ* **1983**, *16*, 306-311.

96. Takita, T.; Hashim, H. Relationship between laboratory-developed biotypes of green leafhopper and resistant varieties of rice in Malaysia. *JARQ* **1985**, *19*, 219-223.
97. Heinrichs, E.A.; Rapusas, H.R. Response to selection for virulence of *Nephotettix virescens* (Homoptera: Cicadellidae) on resistant rice cultivars. *Environmental Entomology* **1990**, *19*, 167-175, doi:10.1093/ee/19.1.167.
98. Dahal, G.; Hibino, H.; Aguiero, V.M. Population characteristics and tungro transmission by *Nephotettix virescens* (Hemiptera: Cicadellidae) on selected resistant rice cultivars. *Bulletin of Entomological Research* **1997**, *87*, 387-395, doi:10.1017/s0007485300037391.
99. Vu, Q.; Quintana, R.; Fujita, D.; Bernal, C.C.; Yasui, H.; Medina, C.D.; Horgan, F.G. Responses and adaptation by *Nephotettix virescens* to monogenic and pyramided rice lines with Grh-resistance genes. *Entomologia Experimentalis et Applicata* **2014**, *150*, 179-190, doi:10.1111/eea.12149.
100. Horgan, F.G.; Bernal, C.C.; Vu, Q.; Almazan, M.L.P.; Ramal, A.F.; Yasui, H.; Fujita, D. Virulence adaptation in a rice leafhopper: exposure to ineffective genes compromises pyramided resistance. *Crop Protection* **2018**, *113*, 40-47, doi:10.1016/j.cropro.2018.07.010.
101. Hirae, M.; Fukuta, Y.; Tamura, K.; Oya, S. Artificial selection of biotypes of green rice leafhopper, *Nephotettix cincticeps* Uhler (Homoptera : Cidadellidae), and virulence to resistant rice varieties. *Applied Entomology and Zoology* **2007**, *42*, 97-107, doi:10.1303/aez.2007.97.
102. Hirae, M.; Tamura, K.; Fukuta, Y. Development and reproduction of biotypes of green rice leafhopper, *Nephotettix cincticeps* (Uhler) (Homoptera: Cicadellidae) virulent to resistant rice varieties. *Japanese Journal of Applied Entomology and Zoology* **2008**, *52*, 207-213, doi:10.1303/jjaez.2008.207.
103. Kaneda, C.; Kisimoto, R. Status of varietal resistance to brown planthopper in Japan. In *Brown Planthopper: Threat to Rice Production in Asia*, International Rice Research Institute, Ed.; International Rice Research Institute: Los Baños, Philippines, 1979; pp. 209-218.
104. Khan, Z.R.; Saxena, R.C. Probing behavior of three biotypes of *Nilaparvata lugens* (Homoptera: Delphacidae) on different resistant and susceptible rice varieties. *Journal of Economic Entomology* **1988**, *81*, 1338-1345, doi:10.1093/jee/81.5.1338.
105. Nemoto, H.; Yokoo, M. Experimental selection of a brown planthopper population on mixtures of resistant rice lines. *Breeding Science* **1994**, *44*, 133-136.
106. Zhao, Y.; Huang, J.; Wang, Z.Z.; Jing, S.L.; Wang, Y.; Ouyang, Y.D.; Cai, B.D.; Xin, X.F.; Liu, X.; Zhang, C.X.; et al. Allelic diversity in an NLR gene *BPH9* enables rice to combat planthopper variation. *Proceedings of the National Academy of Sciences of the United States of America* **2016**, *113*, 12850-12855, doi:10.1073/pnas.1614862113.
107. Guirong, W.; Fengxiang, L.; Qiang, F.; Zhitao, Z.; Lanfang, G. Virulent shift in populations of *Nilaparvata lugens* (Homptera: Delphacidae). *Chinese Journal of Rice Science* **1999**, *13*, 229.
108. Peñalver-Cruz, A.; Arida, A.; Heong, K.L.; Horgan, F.G. Aspects of brown planthopper adaptation to resistant rice varieties with the *Bph3* gene. *Entomologia Experimentalis et Applicata* **2011**, *141*, 245-257, doi:10.1111/j.1570-7458.2011.01193.x.
109. Horgan, F.G.; Ferrater, J.B. Benefits and potential trade-offs associated with yeast-like symbionts during virulence adaptation in a phloem-feeding planthopper. *Entomologia Experimentalis Et Applicata* **2017**, *163*, 112-125, doi:10.1111/eea.12556.
110. Horgan, F.G.; Garcia, C.P.F.; Haverkort, F.; de Jong, P.W.; Ferrater, J.B. Changes in insecticide resistance and host range performance of planthoppers artificially selected to feed on resistant rice. *Crop Protection* **2020**, *127*, 104963, doi:10.1016/j.cropro.2019.104963.
111. Heinrichs, E.A.; Rapusas, H.R. Cross-virulence of *Nephotettix virescens* (Homoptera: Cicadellidae) biotypes among some rice cultivars with the same major-resistance gene. *Environmental Entomology* **1985**, *14*, 696-700, doi:10.1093/ee/14.6.696.
112. Rapusas, H.R.; Chen, J.M.; Heinrichs, E.A. Behavior of two green leafhopper (GLH) colonies on three rice varieties. *IRRN* **1985**, *10-1*, 8.
113. Peng, L.; Zhao, Y.; Wang, H.Y.; Song, C.P.; Shangguan, X.X.; Ma, Y.H.; Zhu, L.L.; He, G.C. Functional study of cytochrome P450 enzymes from the brown planthopper (*Nilaparvata lugens* Stål) to analyze its adaptation to BPH-resistant rice. *Frontiers in Physiology* **2017**, *8*, 972, doi:10.3389/fphys.2017.00972.
114. Gupta, A.; Nair, S. Heritable epigenomic modifications influence stress resilience and rapid adaptations in the brown planthopper (*Nilaparvata lugens*). *International Journal of Molecular Sciences* **2022**, *23*, 8728, doi:10.3390/ijms23158728.

115. Cheng, C.-H.; Chang, W.L. Studies on varietal resistance to the brown planthopper in Taiwan. In *Brown Planthopper: Threat to Rice Production in Asia*, International Rice Research Institute, Ed.; International Rice Research Institute: Los Baños, Philippines, 1979; pp. 251-272.
116. Lee, Y.-M.; Lee, H.-R.; Yi, B.-Y.; Choi, S.-Y.; Sim, J.-W.; Ro, C.-J. Inheritance of adult emergence in artficially induced biotypes of brown planthopper (*Nilaparvata lugens* STAL) on the resistant rice varieties. *Korean Journal of Applied Entomology* **1981**, *20*, 15-20.
117. Tanaka, K. Quantitative genetic analysis of biotypes of the brown planthopper *Nilaparvata lugens*: heritability of virulence to resistant rice varieties. *Entomologia Experimentalis et Applicata* **1999**, *90*, 279-287, doi:10.1046/j.1570-7458.1999.00448.x.
118. Jing, S.L.; Zhang, L.; Ma, Y.H.; Liu, B.F.; Zhao, Y.; Yu, H.J.; Zhou, X.; Qin, R.; Zhu, L.L.; He, G.C. Genome-wide mapping of virulence in brown planthopper identifies loci that break down host plant resistance. *Plos One* **2014**, *9*, e98911, doi:10.1371/journal.pone.0098911.
119. Kobayashi, T.; Yamamoto, K.; Suetsugu, Y.; Kuwazaki, S.; Hattori, M.; Jairin, J.; Sanada-Morimura, S.; Matsumura, M. Genetic mapping of the rice resistance-breaking gene of the brown planthopper *Nilaparvata lugens*. *Proceedings of the Royal Society B-Biological Sciences* **2014**, *281*, 20140726, doi:10.1098/rspb.2014.0726.
120. Pang, R.; Li, S.H.; Chen, W.W.; Yuan, L.Y.; Xiao, H.X.; Xing, K.; Li, Y.F.; Zhang, Z.F.; He, X.L.; Zhang, W.Q. Insecticide resistance reduces the profitability of insect-resistant rice cultivars. *Journal of Advanced Research* **2024**, *60*, 1-12, doi:10.1016/j.jare.2023.07.009.
121. Tang, M.; Lv, L.; Jing, S.L.; Zhu, L.L.; He, G.C. Bacterial symbionts of the brown planthopper, *Nilaparvata lugens* (Homoptera: Delphacidae). *Applied and Environmental Microbiology* **2010**, *76*, 1740-1745, doi:10.1128/aem.02240-09.
122. Wang, W.X.; Zhu, T.H.; Lai, F.X.; Fu, Q. Diversity and infection frequency of symbiotic bacteria in different populations of the rice brown planthopper in China. *Journal of Entomological Science* **2015**, *50*, 47-66.
123. Xu, H.X.; Zheng, X.S.; Yang, Y.J.; Tian, J.C.; Fu, Q.; Ye, G.Y.; Lu, Z.X. Changes in endosymbiotic bacteria of brown planthoppers during the process of adaptation to different resistant rice varieties. *Environmental Entomology* **2015**, *44*, 582-587, doi:10.1093/ee/nvv054.
124. Ojha, A.; Zhang, W.Q. A comparative study of microbial community and dynamics of *Asaia* in the brown planthopper from susceptible and resistant rice varieties. *BMC Microbiology* **2019**, *19*, 139, doi:10.1186/s12866-019-1512-9.
125. Wang, Z.L.; Pan, H.B.; Wu, W.; Li, M.Y.; Yu, X.P. The gut bacterial flora associated with brown planthopper is affected by host rice varieties. *Archives of Microbiology* **2021**, *203*, 325-333, doi:10.1007/s00203-020-02013-8.
126. Xu, X.R.; Chen, L.; Zhou, H.T.; Tang, M. The effect of antibiotic treatment on the bacterial community of the brown planthopper and its correlation with rice virulence. *Agronomy* **2021**, *11*, doi:10.3390/agronomy11112327.
127. Deng, Z.Y.; Lai, C.L.; Zhang, J.; Sun, F.; Li, D.T.; Hao, P.Y.; Shentu, X.; Pang, K.; Yu, X.P. Effects of secondary metabolites of rice on brown planthopper and its symbionts. *International Journal of Molecular Sciences* **2024**, *25*, doi:10.3390/ijms25010386.
128. Horgan, F.G.; Srinivasan, T.S.; Crisol-Martinez, E.; Almazan, M.L.P.; Ramal, A.F.; Oliva, R.; Quibod, I.L.; Bernal, C.C. Microbiome responses during virulence adaptation by a phloem-feeding insect to resistant near-isogenic rice lines. *Ecology and Evolution* **2019**, *9*, 11911-11929, doi:10.1002/ece3.5699.
129. Horgan, F.G.; Peñalver-Cruz, A.; Arida, A.; Ferrater, J.B.; Bernal, C.C. Adaptation by the brown planthopper to resistant rice: A test of female-derived virulence and the role of yeast-like symbionts. *Insects* **2021**, *12*, 908, doi:10.3390/insects12100908.
130. Lai, C.L.; Hou, Y.; Hao, P.Y.; Pang, K.; Yu, X.P. Detection of yeast-like symbionts in brown planthopper reared on different resistant rice varieties combining DGGE and absolute quantitative real-time PCR. *Insects* **2022**, *13*, 85, doi:10.3390/insects13010085.
